# Multimodal (Bio)Markers and Risk of Obesity – A Comprehensive Scoping Review

**DOI:** 10.1016/j.advnut.2025.100579

**Published:** 2025-12-24

**Authors:** Farhad Vahid, Alejandra Loyola-Leyva, Josep Tur, Cristina Bouzas, Yvan Devaux, Laurent Malisoux, Silvia Garcia, Magali De Carvalho, Marina Ródenas-Munar, Jonathan Turner, Elsa Lamy, Maria Perez-Jimenez, Gitte Ravn-Haren, Rikke Andersen, Sarah Forberger, Rajini Nagrani, Maria Giovanna Onorati, Gino Gabriel Bonetti, Daniela Rodrigues, Torsten Bohn

**Affiliations:** 1 Department of Precision Health, Luxembourg Institute of Health, Strassen, Luxembourg; 2 Research Group on Community Nutrition and Oxidative Stress, University of the Balearic Islands-IUNICS (Institut Universitari d'Investigació en Ciències de la Salut), IDISBA (Illes Balears Health Research Institute Foundation) & CIBEROBN (Centro de Investigación Biomédica en Red de la Fisiopatología de la Obesidad y Nutrición), Palma de Mallorca, Spain; 3 Faculty of Science, Technology and Medicine, University of Luxembourg, Esch-sur-Alzette, Luxembourg; 4 Department of Infection and Immunity, Luxembourg Institute of Health, Esch-Alzette, Luxembourg; 5 Mediterranean Institute for Agriculture, Environment and Development & CHANGE (Global Change and Sustainability Institute), University of Évora, Évora, Portugal; 6 National Food Institute, Technical University of Denmark, Kongens Lyngby, Denmark; 7 Leibniz Institute for Prevention Research and Epidemiology—BIPS (Bremen Institute for Prevention Research and Social Medicine), Bremen, Germany; 8 University of Gastronomic Sciences, Bra, Italy; 9 Research Centre for Anthropology and Health (CIAS), University of Coimbra, Coimbra, Portugal

**Keywords:** multicomponent markers, multidimensional, multiclass markers, overweight, miRNA, gut microbiome, diet, emotions

## Abstract

Obesity has been associated with several chronic diseases, especially noncommunicable ones and related comorbidities. Despite international efforts to decrease the prevalence of obesity, the number of persons struggling with this ailment is not decreasing. An important aspect is obesity prevention, including the early detection of the risk, i.e. whether an individual is likely to develop obesity, to allow for early risk stratification and countermeasure initiation. However, obesity is a complex and multifactorial complication, and many factors appear to play a role, including age, sex, diet, physical activity (PA), psychological and emotional status, genetic make-up, epigenetics, and gut microbiota. One isolated biomarker, therefore, could not enable optimal risk stratification and prognosis for the individual; rather, a combined set or multimodal approach to tackle risk prediction is demanded. Such a multimodal interpretation would integrate biomarkers from various domains, such as more classical markers (insulin, leptin), multiomics (e.g. genetics, epigenomics, transcriptomics, proteomics, and metabolomics), behavioral attributes (dietary, PA, and sleep patterns, and smoking status), psychological traits (mental health status, depression, and eating disorders), and gut–microbiota (composition and diversity) into a combined interpretation, also employing more advanced interpretation tools, such as machine learning and artificial intelligence. In this scoping review, we aimed to summarize the current state of the art in this area, highlighting the progress and novel approaches in combating obesity, and focusing on the feasibility and effectiveness of such biomarkers and their application within clinical trials. In addition, we outline potential future steps and recommendations for future approaches.

**Statement of Significance**

This scoping review is the first to comprehensively map and synthesize multimodal (bio)markers across biological, behavioral, and psychological domains for obesity risk prediction, highlighting how integrating these diverse factors, rather than relying on single markers, enhances early detection and informs more effective, personalized prevention strategies. It provides a novel, holistic framework for future research and clinical application, emphasizing the role of nutrition, physical activity, multiomics, machine learning, and objective measurements in advancing obesity risk assessment.

## Introduction

Obesity has become a global public health concern due to its increasing prevalence, its role as a risk factor for multiple other chronic diseases, and its significant socioeconomic impact [1,2]. Overweight and obesity combined were estimated to consume 2.4% of the global GDP in 2020, and are projected to reach 4.32 trillion USD by 2035 [3]. According to the WHO 2022 report, an estimated 60% of the European adult population is having overweight (BMI ≥25 kg/m^2^), with ∼23% having obesity (BMI ≥30 kg/m^2^) [4]. An analysis of obesity trends across 200 countries revealed a significant increase in age-standardized prevalence in adults in 94% of nations between 1990 and 2022 [5].

Obesity is commonly assessed using BMI, waist circumference (WC), body fat percentage, and skinfold thickness [6]. Although these measures are inexpensive and straightforward, they have limitations. For example, BMI does not discriminate between tissue composition, e.g. body fat percentage and its distribution. Advanced methods, such as bioelectrical impedance analysis (BIA) or dual-energy X-ray absorptiometry (DEXA), provide more accurate assessments. Nevertheless, according to the WHO, BMI remains the primary method for classifying obesity [4]; however, specific BMI cutoffs have been recommended for various ethnic groups to improve diagnostic accuracy [7].

A hallmark of obesity, particularly for metabolically unhealthy obesity, is metabolic disturbances, including altered glucose metabolism [8], such as insulin insensitivity and elevated fasting blood glucose levels, increased inflammation (elevated circulating proinflammatory cytokines) [9], and also oxidative stress [10], such as increased levels of F2-isoprostanes. In addition, obesity is a risk factor for numerous noncommunicable diseases, including diabetes [11], cardiovascular diseases (CVD) [12], neurodegenerative diseases [13], inflammatory bowel conditions [14], and several types of cancer [15].

To address obesity’s growing prevalence and its individual and public impact, it is essential to identify early risk factors and improve obesity prognosis. However, due to the complex etiology of obesity and the interaction of multiple host and environmental factors, it is challenging to identify precise mechanisms. Although there is a genetic predisposition for obesity [16], modifiable risk factors such as diet [[17], [18], [19]], physical activity (PA) [20], and sleep quality [21] are crucial in its development. Dietary patterns, including the Mediterranean diet (MD) or other plant-based diets, have been associated with obesity prevention and its management [17,22,23]. For instance, a recent meta-analysis found that greater adherence to the MD was associated with a 9% decreased risk of developing overweight or obesity (relative risk (RR): 0.91; 95% confidence interval: 0.88, 0.94) [24]. However, despite extensive research on the causes and consequences of obesity, its etiology remains challenging to understand due to its complex multifactorial nature [25].

Single (bio)markers may thus inadequately capture the risk of developing obesity. Consequently, recent efforts have shifted toward combining multiple individual factors into more comprehensive risk scores. Such multicomponent (bio)markers, i.e. integrating markers from 1 domain, or multimodal markers, i.e. combining markers across different domains, for obesity risk prediction, could include combining several individual markers, e.g. blood pressure, biochemical parameters (e.g. blood cell profiles, fasting glucose, etc.), inflammatory biomarkers, and oxidative stress. More novel approaches, such as genetic markers [e.g. single-nucleotide polymorphisms (SNPs)], epigenetic modifications including methylation patterns or miRNAs, transcriptomic markers (RNA expression profiles and RNA modifications), gut microbiota diversity (e.g. α-or β-diversity or Firmicutes:Bacteroidetes ratio [26]), which multiomics approaches may measure, are also emerging [27,28]. These biomarkers may be combined with lifestyle factors such as nutrient intake or status [6,16,26,29,30], PA, including sedentary behavior [[31], [32], [33]], sleep quality [34,35], and emotional well-being [[36], [37], [38]] into multimodal (bio-)markers for a more comprehensive modeling to assess obesity risk.

The emerging field of multicomponent (bio)marker analysis [39] presents a promising approach to understanding the interactions between biological and lifestyle factors and the development of obesity [40,41]. For example, genome-wide association studies (GWAS) have identified genetic variants linked to obesity and body fat distribution, including the fat mass and obesity (FTO) gene [42,43]. Although genetic factors, according to twin studies explaining heritability, may explain 40% to 70% of individual variation in BMI [44], recently developed polygenic risk scores have explained a much smaller variability of BMI in the general population, with a genetic risk score (GRS, based on some SNPs), i.e. <2%, as expressed by correlation coefficients [45]. However, diet, PA, and socioeconomic status often play a more substantial role in determining actual weight outcomes. Thus, although GRSs offer some predictive value, they remain part of a broader, multidimensional understanding of obesity, where genetics, environment, and lifestyle intersect [46,47].

More advanced approaches with multimodal biomarkers may integrate genetics and epigenetics to diagnose specific diseases. Such an approach has been used to accurately (96.4%) diagnose psoriasis vulgaris, using a support vector machine-based classification model employing the expression levels of 4 genes (*IL36G, CCL27, NOS2, and C10orf99*) [48]. Other studies, such as those related to bioactive plant compounds, have, for example, predicted outcomes of carotenoid metabolism by combining SNPs and epigenetics, accounting for ≤73% of the variability in the bioavailability of lutein [49]. Combining genetic and epigenetic changes with lifestyle aspects is expected to enhance predictability compared with individual markers.

Thus, such multimodal approaches may offer more robust predictions, providing a cumulative perspective on the impact of biomarkers and assessing their association with obesity across different age groups more accurately. Additionally, using multiple biomarkers might well explain individual variability, improve the diagnosis of related diseases, and create predictive models that estimate both population and individual risk. This approach has been employed in computational methods, including machine learning algorithms and model-based prediction [48,50,51]. Understanding the most critical risk factors (and their interaction) associated with obesity development is essential for developing effective evidence-based interventions and formulating impactful public health policies targeting prevention and reducing health complications.

To our knowledge, no comprehensive review currently addresses multimodal (bio)markers and their association with obesity risk. Existing systematic reviews have focused on workplace-related multimodal interventions [52] and individual multimodal interventions for children, adolescents [53,54], and adults with obesity [55], with limited emphasis on early risk detection and prevention. Therefore, this scoping review aims to explore, map, and summarize current approaches in multimodel biomarkers related to obesity prediction. It will help provide a more holistic understanding of obesity’s complex nature, encompassing a range of biological and nonbiological factors, and highlight recent advancements in the field.

## Methods

### Review protocol, outcome definition, and defining multicomponent biomarkers

The protocol for this scoping review has been published previously [56]. For the methodology design, the Open Science Framework guidelines were followed [57], and the study was registered on the platform (DOI: https://doi.org/10.17605/OSF.IO/4WT9X). In addition, it followed the PRISMA guidelines and the extension for scoping reviews (PRISMA-SCR and flowchart) [58]. The approach was structured based on Arksey and O’Malley’s scoping review methodology [59], with methodological adaptations by Levac et al. [60].

Regarding the outcome of obesity, we followed the definition provided by the authors, which typically included definitions based on BMI (>30 kg/m2), visceral body fat, percentage fat mass, among other.

Many terms for combining multiple biomarkers are used in the literature, including multicomponent, multiclass, and multidimensional. However, we adopted the term “multimodal” (Table 1) in alignment with recent consensus reports and methodological frameworks in systems and obesity research, emphasizing the diverse domains from which the (bio)markers were derived. Furthermore, we refer in this text to the term (bio)marker, to summarize indicators of biological processes or responses to exposure/interventions that may include a variety of endpoints, such as molecular, histologic, or physiological characteristics, similar as stated by the NIH and the FDA [61]. Supplemental Figures 1 and 2 illustrate the detailed workflow of study selection and the mapping process of multimodal (bio)markers across study types.

**TABLE 1:** Definition of multimodal biomarkers (what is/is not considered multimodal) in this article

Independent components	Examples for each component
Diet	Nutritional aspects (plasma/serum nutrients, serum albumin, pre-albumin, urine concentration of nutrients), FFQ, 24-h recalls, dietary records, other questionnaires, dietary supplements
PA behavior	PA, 24-h movement, sedentary behavior (including sitting, TV time), METs, sleep, video gaming, etc.
Mental	Emotions/psychology/mental aspects/cognitive (stress, IQ)/behavior
Omics	Transcriptomics/proteomics/metabolomics, gut microbiota (meta-transcriptomics, 16S RNA, etc.)
Genetics	Genetics and epigenetics
Traditional markers	Blood, urine, and hair markers such as blood lipids, glucose, cytokines, cell counts, oxidative stress, hormones, and blood pressure (also arterial stiffness, central BP, etc.).
✓ Yes—combining any of ≥2 independent components^1^ Examples of component combinations: a) Diet and PA behavior: serum nutrients, and sedentary behavior b) Traditional markers and Omics: cytokines and 16S RNA c) Genetics and omics: SNPs and gut microbiota d) PA behavior and genetics: 24-h recalls and epigenetics e) Traditional markers and PA behavior: blood pressure and TV time f) Diet and PA behavior: vitamin D and sleep g) Mental and omics: cognitive and transcriptomics h) Mental and traditional markers: emotions and hormones	
✘ Not—including only 1 independent component or 2 measurements from the same independent component^2^ Examples of combinations not qualifying: Diet: a) 2 nutritional aspects, that is, intake of proteins and micronutrients b) FFQ and plasma minerals c) dietary index and dietary questionnaires d) supplementation with vitamin D and diet quality PA:PA and sedentary behaviorMental:Mental health and cognitive aspects aloneOmics: a) plasma proteomics and saliva proteomics b) transcriptomics and proteomics c) gut microbiota measured by various methods d) females vs. males and gut microbiota Traditional markers: a) 2 classical biomarkers in the bloodstream, that is, inflammation and blood glucose b) systolic blood pressure (SBP) and blood glucose Others^3^ a) surgery (bariatric or digestion-related), drugs/medication (under the EU pharma regulation) b) any study involving drugs also in combination c) any study not clearly showing that an obesity-relevant endpoint was measured	

Abbreviations: BP, blood pressure; EU, European Union; FFQ, food frequency questionnaire; METs, metabolic equivalents; PA, physical activity; SBP, systolic blood pressure; SNPs, single-nucleotide polymorphism.

1 That is, the presence of ≥2 combinations of the following independent measures listed in different rows.

2 Note that not counted as a risk factor are anthropometrics (weight, height, hip-waist ratio, thigh circumference, body fat distribution, and visceral fat); these are considered as outcomes (measures of obesity).

3 Considered as treatment in the medical field are not included here.

### Eligibility criteria

The inclusion and exclusion criteria are described in Table 2. Case series, reports, studies in languages other than English, and those involving animal or cellular models were excluded to facilitate more homogeneous data interpretation and ensure higher evidence quality, that is, based on high-quality human studies [62]. We focused on biological and behavioral (bio)markers directly related to obesity risk. Although environmental and socioeconomic factors are recognized as important contributors to obesity, these domains were intentionally excluded as their scope was deemed too broad and complex to be meaningfully integrated within the (bio)marker framework applied here.

**TABLE 2:** Summary of inclusion and exclusion criteria [56]

Aspect	Inclusion	Exclusion
a) Literature	Original peer-reviewed research papers, systematic reviews, meta-analyses, reviews	Gray literature, abstracts, PhD theses, editorials, books, project reports, non-reviewed conference proceedings
b) Main outcome/health complication	Risk of overweight and obesity and management and all markers thereof: PA, diet, socioeconomic aspects, host factors such as genetics, epigenetics.	Studies not related to the risk of overweight and obesity.
c) Population	All populations, all ages, later mapping to target groups (older adults ≥65 y, children (5–12 y), young adults (18–25 y)	—
d) Region	Whole world	—
e) Studies to be appraised for quality	Intervention, observational (case-control, prospective…), pooled data	Case reports, case series
f) Time	All through December 2023	—
g) Language	English (abstract and whole text)	Non–English (abstract and whole text)
h) Species	Human studies (both sexes)	Animal studies, cellular models, and in vitro studies

Abbreviation: PA, physical activity.

### Information sources and search strategy

Four major databases—PubMed, Embase, Scopus, and CINAHL—were used for the search, which has been published elsewhere [56]. The electronic search strategy was developed in collaboration with all authors, based on their specific expertise. The search was conducted using an initial set of search terms. An experienced research librarian was involved in the following aspects of the search syntax development: 1) translating research questions into search terms, 2) appropriate use of adjacency proximity operators, 3) text word and mesh-term searching done by inspecting the truncation and inclusion of British and American spellings, and 4) errors in spelling and syntax were corrected by meticulously reviewing each line of the syntax strategy and checking the application of parentheses and Boolean operators.

Two team members carried out the search and extraction of articles from the databases. The articles were exported to EndNote 21 (Clarivate Analytics), which was also used to deduplicate findings from the databases. Following this step, articles were transferred to the free, web-based CADIMA tool for further study screening and selection. The procedure adhered to the Peer Review of Electronic Search Strategies guideline [63].

### Study selection/screening

**FIGURE 1:**
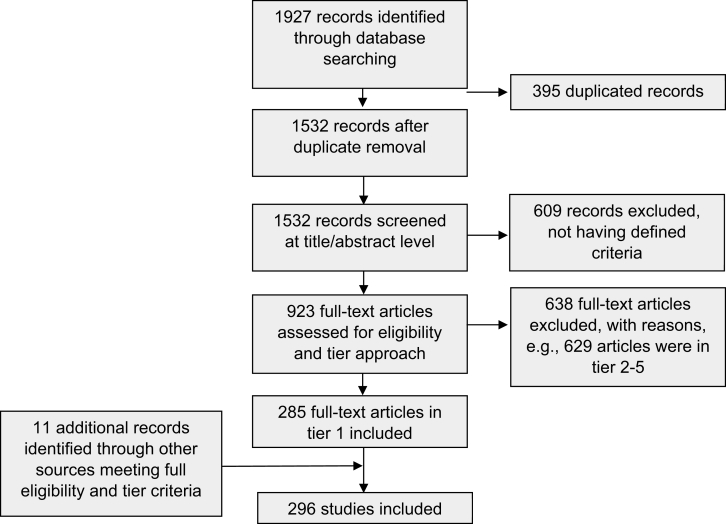
PRISMA flow diagram of study selection and screening process.

Initially, titles and abstracts of potential studies were screened for relevance. A pilot screening phase was conducted to ensure consistency and alignment among the screening team members. Two reviewers (FV, TB) performed the screening independently. Any discrepancies or disagreements in the screening process were addressed through discussion and consensus among the reviewers and the other authors. The articles initially identified were screened for thematic fit (the relevance of the articles to the main focus or theme of the study) after removal of the duplicates. If the information in the titles and abstracts was unclear, additional context or full-text examination was used to make an informed decision. CADIMA was utilized to manage and document the title, abstract, and full-text screening process, as well as data extraction. After title and abstract screening, full texts of selected studies were thoroughly examined to assess their eligibility (Figure 1).

### Data collection and extraction

First, a pilot phase was completed to refine the approach and achieve consistency. A selected subset of studies or sources was utilized for practice and feedback. Two team members extracted the data independently of each other. Additional team members critically reviewed the extracted data. Inconsistent terminology was harmonized, and incomplete data in the articles were strived to be retrieved by additional published sources or by contacting the authors if required. Excel, Word, and SPSS were used for analysis and data summarization. Discrepancies were resolved during regular team meetings as well as the involvement of an arbitrator or lead reviewer when consensus was elusive.

Furthermore, a tiered approach was employed to classify articles into 5 tiers (Supplemental Table 1). The articles in tiers 2 to 4 were used for general review, state-of-the-art, introduction, and discussion. Data from articles in tier 1 containing quantitative data relating to the main outcome, weight change risk, with ≥2 eligible components (e.g. PA, and diet, see list, n = 296) were extracted and used for mapping and analysis. Detailed information for all studies included in this review is provided in the Supplemental Excel Table 2.

The results were mapped based on the components, categorizing the articles into 9 distinct groups (Table 3) to organize results and discussions.

**TABLE 3:** The overall results of the studies are included in the scoping review mapping

Components	Articles in groups (total n = 296)^1^	Articles with significant results^2^	Articles with nonsignificant results^3^	Type of sign. finding (as= association, co= correlation, ch= change)^4^	Fraction of significant to all articles (b/a)^5^
Anywhere					
Diet	273	226	44	as= 98, co= 33, ch= 160	0.82
PA	194	165	28	as= 63, co= 22, ch= 121	0.85
Omics	67	56	11	as= 31, co= 13, ch= 37	0.83
Genetics	52	40	11	as= 26, co= 5, ch= 24	0.77
Traditional markers	120	99	20	as= 45, co= 23, ch= 68	0.82
Mental health	116	98	16	as= 37, co= 10, ch= 74	0.84
Combinations					
Group 1 (diet, PA): a. diet, PA (n = 39)	39	31	8	as= 7, co= 2, ch= 25	0.79
Group 2 (diet, PA, mental): a. diet, PA, mental (n = 58)	58	50	8	as= 20, co= 1, ch= 37	0.86
Group 3 (diet, PA, any other): a. diet, PA, traditional markers (n = 26) b. diet, PA, omics (n = 8) c. diet, PA, genetics (n = 6) d. diet, traditional markers (n = 8)	48	43	4	as= 19, co= 13, ch= 26	0.89
Group 4 (diet, omics, any other): a. diet, omics (n = 24) b. diet, omics, traditional markers (n = 16) c. diet, genetics, omics (n = 3)	43^6^	35	8	as= 21, co= 10, ch= 19	0.81
Group 5 (diet, genetics, traditional markers): a. diet, genetics (n = 13) b. diet, genetics, traditional markers (n = 11)	24	16	8	as= 10, co= 1, ch= 9	0.66
Group 6 (diet, mental, any other): a. diet, mental (n = 10) b. diet, mental, traditional markers (n = 7) c. diet, mental, genetics (n = 1)	18	12	4	as= 5, co= 1, ch= 10	0.66
Group 7 (PA, no diet): a. PA, traditional markers (n = 4) b. PA, omics (n = 2) c. PA, genetics, traditional markers (n = 1) d. PA, mental, traditional markers (n = 1) e. PA, genetics (n = 1) f. PA, mental (n = 7)	16	12	4	as= 4, co= 6, ch= 4	0.75
Group 8 (no diet, no PA) a. genetics, traditional markers (n = 4) b. mental, traditional markers (n = 2) c. mental, genetics (n = 1)	7	5	2	as= 4, co= 1, ch= 2	0.71
Group 9 (any 4): a. diet, PA, mental, traditional markers (n = 22) b. diet, PA, omics, traditional markers (n = 6) c. diet, PA, genetics, omics, traditional markers (n = 3) d. diet, PA, genetics, traditional markers (n = 4) e. diet, PA, mental, omics (n = 2) f. diet, PA, mental, genetics, traditional markers (n = 2) g. diet, PA, mental, omics, traditional markers (n = 1) h. diet, mental, omics, traditional markers (n = 1) i. diet, genetics, omics, traditional markers (n = 1) j. diet, PA, mental, genetics (n = 1)	43	39	4	as= 16, co= 5, ch= 34	0.91

Abbreviations: as, association; ch, change; co, correlation; PA, physical activity.

1 All articles included in this review.

2 At least one of the obesity-related outcomes (primary outcome) was significant.

3 None of the obesity-related measurements were significant.

4 The total count may exceed the number of articles in the group, as some studies investigated multiple aspects.

5 Fraction of articles with significant findings vs. overall articles.

6 In 1 article, they did not specify whether the result was significant.

## Results

### Overall findings

#### Studies, design, participants, and main outcomes

**FIGURE 2:**
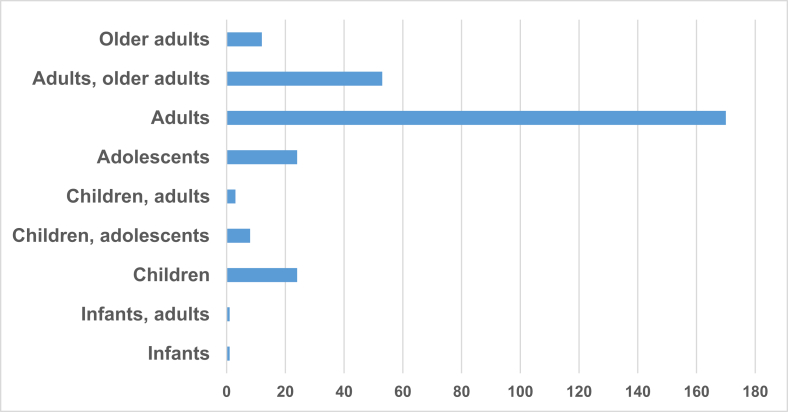
Age distribution of participants across the included studies according to the number of articles.

**FIGURE 3:**
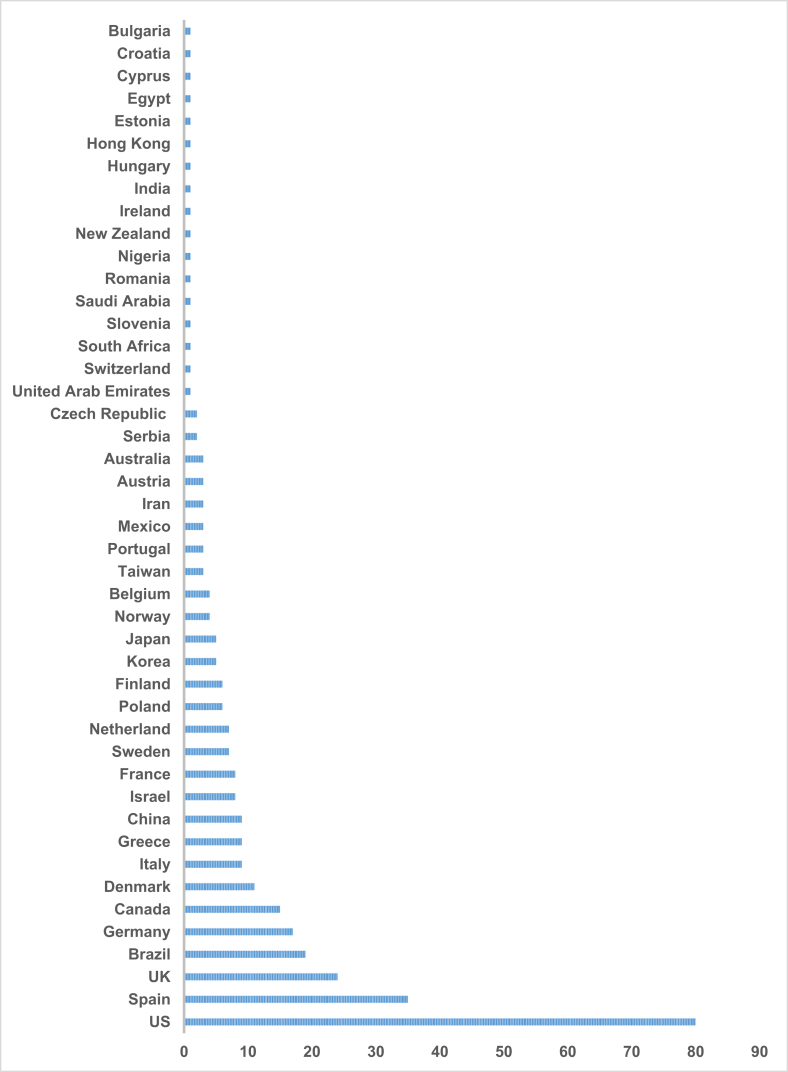
Geographic distribution of studies across countries.

Of the 296 articles included in the review, the majority of studies (85.5%) focused on adults and older adults (Figure 2). Additionally, 76% of the studies included both sexes, 4% focused exclusively on males, and 20% on females. All studies collectively examined a total of 554,901 participants. The mean sample size was 1900, with a median of 94, a minimum of 10 participants, and a maximum of 378,877 participants (Supplemental Table 2 and 4). 21.6% of the studies focused on healthy individuals, 56.4% on individuals with overweight or obesity, and 8.0% on individuals with metabolic syndrome (MetS). The years with the highest number of articles published were 2021 (37 articles) and 2019 (30 articles) (Supplemental Figure 3). The United States (80 articles), Spain (35 articles), and the United Kingdom (24 articles) are the countries that showed the most focus on the topic (Figure 3).

**FIGURE 4:**
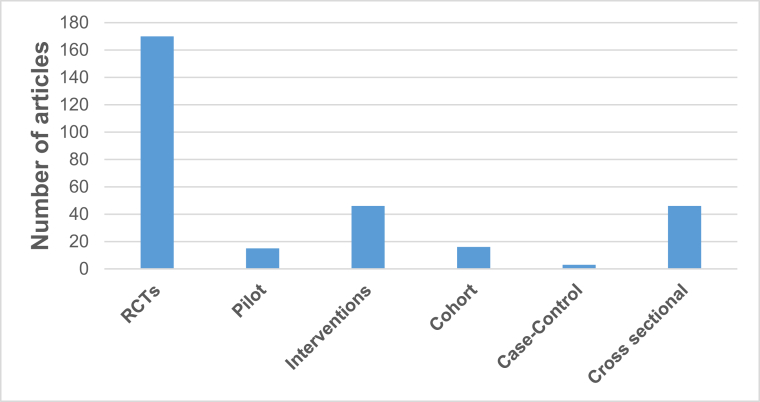
Distribution of studies based on their design. The intervention studies include all studies that have not been conducted in clinical trial settings. Pilot studies encompassed different study designs, including RCTs. RCT, randomized controlled trials.

The majority of studies (57.4%) had a randomized controlled trial (RCT) design (Figure 4), whereas 15.5% were cross-sectional. In this review, we considered both “association” and “correlation” as broadly indicating relationships between variables, in contrast to “effect” which typically refers to interventional evidence. Although we acknowledge that correlation represents a specific statistical measure, we retained the original wording used by the authors for consistency with the source publications. Most studies (60.0%) used only anthropometric methods to measure outcomes, 36.8% used a combination of anthropometric methods and other techniques, such as DEXA, BIA, etc., whereas only 2% of studies employed nonanthropometric methods.

#### Components and (bio)markers

**FIGURE 5:**
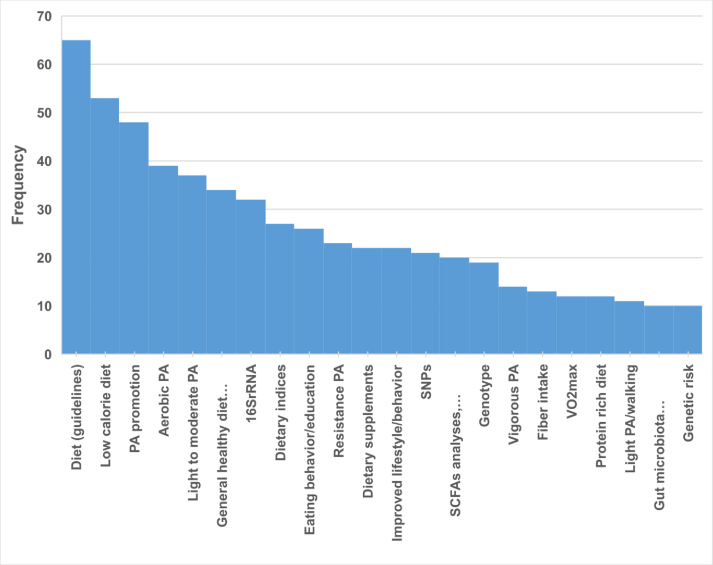
Distribution of the most frequent aspects, here termed as (bio)markers, assessed in the studies. Only categories with a frequency of 10 or more observations are presented. PA, physical activity; SNPs, single-nucleotide polymorphisms.

The articles identified several (bio-)markers or aspects used to measure the effect of each main component of the study (Figure 5). For example, regarding the dietary component, giving general dietary guidance and prescribing a low-caloric diet (with various definitions, though typically following a reduction of, e.g. 20% to 30% from baseline intake) were most frequently associated with obesity-related outcomes. For PA, general PA promotion (of a broad measure, but often suggesting 150 min of moderate-intensity PA per week, which translates to ∼30 min/d, 5 d a week) and light-to-moderate PA recommendations were related to weight-related improvements. Regarding mental health, lifestyle improvements and cognitive-behavioral aspects were mostly investigated, followed by perceived stress and cognitive/behavioral therapies. For the traditional markers, lipid profiles and hormonal markers were the most frequently assessed, followed by glucose homeostasis and antioxidants. Within the field of –omics-related biomarkers, gut microbiota analyses by 16S rRNA were the most extensively explored biomarker analysis, followed by analyses of short-chain fatty acids and metabolites. Within genetics, SNPs and genotype analysis were the most prominent potential biomarkers studied.

#### Statistically significant findings

Regarding statistical analyses, the most prevalent intervention studies included Student’s t-tests and analysis of variance (ANOVA), though also more refined models such as linear mixed models. For observational investigations, regression and correlation statistics (chi-square tests, Pearson, and Spearman) were most often employed. These reflect a strong focus on comparing means, analyzing relationships between variables, and modeling data with complex or hierarchical structures. Principal component analysis (n = 11) and machine learning methods (n = 2) were also only used in some analyses.

**FIGURE 6:**
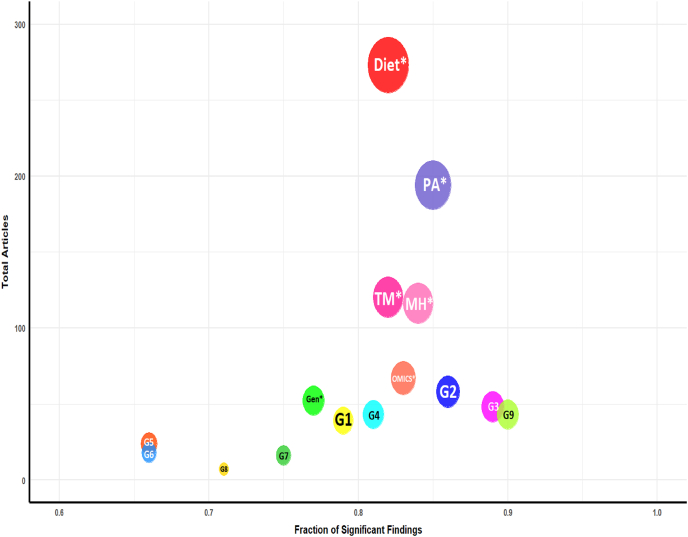
Balloon chart of overall results showing the fraction of significant findings of components compared with all articles retrieved in the respective category, compared with the total number of articles with that component. The size of the circle also represents the number of articles. ∗Individual components appearing anywhere, for example, diet with a combination of ≥1 other component. Gen, genetics; G1, group 1 (diet, PA); G2, group 2 (diet, PA, mental); G3, group 3 (diet, PA, any other); G4, group 4 (diet, omics, any other); G5, group 5 (diet, genetics, any other); G6, group 6 (diet, mental, any other); G7, group 7 (PA, no diet); G8, group 8 (no diet, no PA); G9, group 9 (any 4); MH, mental health; PA, physical activity; TM, traditional markers.

Regarding individual components, the highest proportion of statistically significant findings with obesity-related outcomes (i.e. ≥1 of the primary outcomes reporting a significant finding), relative to the total number of articles examined, corresponded to studies incorporating PA (0.85), mental health (0.84), and omics (0.83), in combination with any other components (Figure 6).

Multimodal groupings revealed that group 9 (studies incorporating >3 components) exhibited the highest proportion of statistically significant findings relative to the total articles within this group (0.91). Thus, integrating multiple health domains may offer a more comprehensive assessment of obesity determinants and may increase the likelihood of detecting significant findings, including clinically relevant ones (Table 3); however, the logistic complexity of such designs may limit their applicability in certain contexts. Additionally, group 3, combining diet, PA, and 1 additional component, and group 2, which includes diet, PA, and mental health, ranked second and third in significant result proportions, at 0.89 and 0.86, respectively. Conversely, group 5 (diet, genetics, and 1 additional component), and group 6 (diet, mental health, and 1 additional component), displayed the lowest proportions of statistically significant findings (0.66 in both cases). These insights provide guidance for optimizing study design, suggesting that combinations incorporating PA, diet, and mental health not only yield high research impact but also offer practical advantages for advancing our understanding of the multifaceted contributors to overweight and obesity. Detailed interpretations and implications of these findings are presented and discussed in the corresponding sections.

### Group 1: diet and PA

#### Overview of articles retrieved, type of studies, number of combinations, and main findings

A total of 38 studies combining diet and PA either as exposure or intervention were found. The articles were primarily from Europe (n = 15) [[64], [65], [66], [67], [68], [69], [70], [71], [72], [73], [74], [75], [76], [77], [78]] and the United States (n = 13) [[79], [80], [81], [82], [83], [84], [85], [86], [87], [88], [89], [90], [91]]. The sample size ranged from 10 to 6217 (mean 349) participants. Most studies were conducted with adults (n = 23), 5 in adults and older adults, 3 in older adults, 2 in young adults and 5 in children. The ages included in each population group were very heterogeneous and did not appear to follow any specific pattern. Finally, most articles included both sexes; however, 2 papers included only males, and 7 papers only females. The 38 articles included mainly RCTs (n = 21) [64, [66], [67], [68], [69], 73, 74, [77], [78], [79], [80], [81], 83, 84, 86, 87, [91], [92], [93], [94], [95]] and other intervention studies (n=11) [[70], [71], [72], 75, 76, 82, [88], [89], [90], 96, 97]. The other were longitudinal and cross-sectional studies, which complemented this research by offering both temporal insights and providing overviews of population data.

The interventions combined nutrition education, PA, and behavioral strategies related to diet and PA, such as goal setting, self-monitoring, and problem-solving to encourage weight loss and improve health outcomes. The use of multimodal approaches (diet, exercise, including, in part, and behavioral support) seemed to offer the most comprehensive solution for achieving and maintaining weight loss (Figure 6). Intervention durations ranged from 4 d to a 24 mo.

#### Discussion on studies combining diet and PA and measured markers

Regarding the observed (bio-)markers, dietary changes were frequently monitored through self-reported food frequency questionnaires (FFQs), 24-h dietary recalls, or dietary records [64, 65, [68], [69], [70], [71], [73], [74], [75], 78, 83, [89], [90], [91], 93, 94, 96, [98], [99], [100]], often focusing on caloric intake, macronutrient composition (particularly fat and sugar intake), adherence to specific dietary patterns such as the MD or low-calorie diets, and assessment of satiety, appetite or satisfaction among other food-related feelings. Regarding PA and fitness/performance, changes were typically assessed through self-reported questionnaires (e.g. IPAQ), accelerometry, or via performance-based tests, such as grip strength, timed up-and-go, or aerobic capacity tests (e.g., VO_2_max or submaximal exercise tests) [65, 66, [69], [70], [71], [72], [73], [74], 78, 79, [90], [91], [92], 95, 96, 98, 99, 101]. In some studies, (bio-)markers of muscle strength or cardiorespiratory fitness [66, 79, 101 were also considered.

In most studies, the investigated outcomes/endpoints were BMI, body weight, WC, and body composition. BMI was the most commonly employed indicator of obesity, offering a fast and simple measure to judge overall body fat. Other frequently measured markers included WC and waist-to-hip ratio (WHR), which help evaluating abdominal fat distribution and body composition, particularly the assessment of fat mass and lean mass, which provides a more detailed understanding of an individual’s health status. Especially WC could be considered a simple and potentially strong predictor of obesity risk that can be implemented in both research and clinical settings [[64], [65], [66], 68, 70, 71, 73, 74, 81, [91], [92], [93], [94], [95], 98, 99, 101]. It is a quick, low-cost, and reliable measure of central adiposity, which is strongly associated with metabolic risk and future weight gain [102, 103]. Within body composition, 7 articles mainly focused on body fat [64, 68, 71, 73, 86, 96, 98], one focused on bone marrow adipose tissue [98], and another one on abdominal adiposity and liver fat content [64]. Only 2 papers included hip circumference [65, 66], and 1 bone mineral density (BMD) [79]. Measures such as the Fat Mass Index, expressed as kilograms per square meter [104] could further complement the analysis. Such endpoints can contribute valuable insights into the metabolic effects of excess fat, particularly in relation to organ-specific fat accumulation. The above markers, when considered together, provide a more comprehensive assessment of an individual’s health compared with e.g. only BMI, offering insights into not only obesity but also potential risks for related conditions, such as MetS, CVD, and diabetes. The use of these markers, especially when further combined with lifestyle factors such as diet and PA, can be crucial for identifying individuals at risk and developing targeted prevention or intervention strategies.

Endpoints were measured by anthropometric measurements or bioimpedance analysis (BIA), and in some cases, DEXA [66, 69, 70, [79], [80], [81], 86, 96, 101] or MRI [64, 98]. Most articles (31/38) showed significant results in health-related biomarkers, e.g. functional exercise capacity, VO2max, and dietary indices. In addition, grip strength or muscle strength tests [105, 106], adjusted for body size, could be promising functional markers, as lower muscular fitness in youth has been linked to higher fat mass and metabolic dysfunction [105, 106]. Moreover, submaximal aerobic tests (e.g. step test or shuttle run) can also be useful for assessing cardiorespiratory fitness, which inversely correlates with adiposity [[107], [108], [109]]. Many studies (n = 23) evaluated only the change of the variables studied, 5 evaluated only the association between the variables, 1 assessed changes and associations, 1 analyzed changes and correlations, and only 1 assessed changes, associations, and correlations.

Nutritional benefits were particularly evident when a healthy diet was controlled, enhancing both physical well-being and metabolic health [83, 85, 94]. However, one study reported problems with weight loss, showing that even combined with exercise, it might decrease bone mass density in older adults with obesity [84], which is a critical aspect to be avoided, as well as muscle mass loss, as both are related to sarcopenia [110]. Moreover, psychological aspects [68] and environmental influences, such as urbanization [99], on weight management were mentioned, though these aspects appeared less frequently in the literature, and the environment was beyond the scope of this review. Similarly to the current findings, a previous systematic review and meta-analysis provided evidence of the benefits of combining PA and diet rather than promoting these therapies individually in weight management programs, also showing greater effects in multimodal interventions [111].

In conclusion, many studies emphasized the positive effects of combining PA and a healthy diet, resulting not only in notable weight management outcomes but also in a wide range of benefits for several conditions, such as cancer, CVD, and osteoarthritis [[112], [113], [114], [115], [116], [117], [118], [119], [120], [121], [122], [123], [124], [125]]. These benefits may also be achieved through diet or PA intervention alone [119, [126], [127], [128], [129], [130]], but the results showed greater promise when both were combined.

#### Limitations

Despite several studies using objective measures, such as accelerometers or bioimpedance analysis, subjectivity in self-reported endpoints, including questionnaires (IPAQ, 24-h recalls, etc.), remains a challenge, impeding the accuracy and precision of the determined endpoints. Objective data collection methods, such as accelerometers for PA measurement [131, 132] and biomarkers of intake for nutritional endpoints [133], should be prioritized to reduce bias. The variability in methodologies across studies may hinder direct comparisons of results, as differences in study design, population characteristics, and measurement techniques can lead to divergent findings. For example, some studies focused on specific age groups, such as children or older adults, whereas others included a more heterogeneous sample (age, sex, etc.), which may have influenced the effectiveness and outcomes of interventions. A common subject that was also stressed was the importance of intervention strategies [64, 67, 71, 73, 75, 82, 88, 90, 93, 96, 97, 101] and the need for a multifaceted approach that goes beyond PA and dietary adjustments alone, incorporating cultural, community, and also individual factors to ensure a more comprehensive and sustainable impact on weight management. One of the latest additions to these strategies that has shown promise is the use of e-health technologies, which can enhance engagement and provide personalized support for individuals [88, 90, 96].

### Group 2: diet, PA, and mental health

#### Overview of articles retrieved, type of studies, number of combinations, and main findings

A total of 58 articles reported on PA and mental health related to eating behavior. Most of these studies originated from the United States (n = 21) [[134], [135], [136], [137], [138], [139], [140], [141], [142], [143], [144], [145], [146], [147], [148], [149], [150], [151], [152], [153], [154]], followed by Spain (n = 7) [124, [155], [156], [157], [158], [159], [160]], Germany and Brazil (n = 6 each) [112, 116, [161], [162], [163], [164], [165], [166], [167], [168], [169], [170]], the United Kingdom (n = 4) [[171], [172], [173], [174]], and Norway (n = 2) [53, 175]. One article reported findings from a multicountry study [176]. Results were most frequently reported for adult populations (n = 23) [55, 112, 126, 135, [138], [139], [140], [141], [142], 144, [147], [148], [149], 153, 159, 167, 170, 172, 173, [177], [178], [179], [180]], followed by adults and older adults combined (n = 19) [136, 137, 143, 145, 146, 154, 155, 157, 160, 164, 171, [174], [175], [176], [181], [182], [183], [184], [185]], adolescents (n = 8) [134, 151, 152, 158, [161], [162], [163], 166], and children, as well as children and adolescents combined (n = 6 [116, 124, 156, 165, 168, 169], n = 2 [53, 100]). Most studies (n = 49) presented findings for both sexes, whereas 9 studies focused exclusively on females. Nine studies reported findings for healthy individuals, 9 focused on overweight individuals, and 7 on those with overweight or obesity. The remaining articles concerned individuals with various health characteristics, such as type 2 diabetes, overweight, prediabetes, schizophrenia, or MetS. The chosen study designs included RCTs (n = 35) [53, 100, 112, 124, 126, [134], [135], [136], [138], [139], [140], [141], [143], [144], [145], 149, [151], [152], [153], 156, 157, [159], [160], [161], 168, [170], [171], [172], [173], [175], [176], [177], [178], [179], 184], nonrandomized intervention studies (n = 11) [116, 142, 146, 148, 158, [162], [163], [164], 167, 169, 180], cross-sectional observational studies (n = 7) [154, 165, 166, 171, 174, 181, 182], prospective cohort studies (n = 3) [55, 155, 183], and pilot RCTs (n = 2) [137, 185]. The duration of interventions varied from 4 wk to 11 y, but mostly lasted 4 wk to 6 mo (n = 11), followed by those lasting 6 mo to 1 y (n = 6) and 1–11 y (n = 6). Four studies reported follow-up data collection [143,167,171,186].

#### Discussion on studies combining diet, PA, and mental components, and measured markers

In total, the articles report 42 different (bio)markers. The most frequently reported (bio)marker was “PA promotion” (n = 14) [124, 151, 153, [155], [156], [157], 160, 161, 164, 165, [170], [171], [172], 179, 185], followed by “eating behavior and education” (n = 10) [112, 116, 124, 137, 139, 152, 169, 170, 176, 185], “light to moderate physical activity” (n = 9) [137, 139, 141, 143, 145, 148, 149, 177, 182] and “diet based on guidelines” (n = 9) [135, 136, 143, 145, 146, 154, 157, 160, 176], “low calorie diet” (n = 8) [100, 141, 142, 148, 154, 159, 177, 183]. Eight studies did not report on any (bio)markers [53, 134, 144, 147, 158, 168, 178, 181].

The combination of the 3 (bio)markers (very low calorie diet (VLCD), improved lifestyle and behavior, dietary supplement intake) was reported by 5 studies [55, 167, 174, 175, 184]. Fourteen studies reported on 2 (bio)markers [112, 116, 138, 141, 148, 154, 161, 164, 169, 172, 173, 179, 180, 183] in various combinations, where the combination of “PA promotion,” “general healthy diet promotion” was reported n = 4, and “vigorous PA,” “eating behavior and education” n = 2 times [116, 169]. Eight articles reported [137, 139, 142, 145, 156, 165, 170, 185] 3 (bio)markers, and all other studies >3 (i.e., 4 (bio)markers n = 14, 5 (bio)markers n = 5, 6, and more (bio)markers n = 4) [100, 124, 126, 135, 136, 140, 143, 146, 149, [151], [152], [153], 155, 157, 159, 160, 162, 163, 166, 171, 176, 182].

Combinations of (bio)markers varied greatly. The most frequent one was “PA promotion,” “general healthy diet promotion,” appearing 4 times on its own and 6 times in combination with other (bio)markers [151, 156, 161, 164, 165, [170], [171], [172], 179, 185]. The combination of “light to moderate physical activity,” “low calorie diet” was reported 3 times [141, 148, 177]. Other combinations were “vigorous PA” and “eating behavior and education” (n = 2; [116, 169] and “resistance PA,” “aerobic PA,” combined with other markers (n = 3 [162, 163, 166].

In most of articles (n = 40), the outcomes were anthropometry, whereas in 2 of these studies (n = 2) they were self-reported [174, 182]. In n = 17 studies, anthropometry was combined with other outcomes such as DEXA, BOD POD (air displacement plethysmography), body composition, and bioelectrical impedance [112, 135, 137, 140, 141, 143, 149, 158, 159, 162, 163, 165, 171, 173, 178, 179, 185]. One study did not report on outcome measurements [53].

Of the 56 articles reporting on anthropometric measures, the most commonly reported outcome was BMI (n = 55) or, in combination with weight loss, body composition, WC, or fat mass [55, 112, 116, 124, 126, [134], [135], [136], [138], [139], [140], [141], [142], [145], [146], [147], [148], [149], [151], [152], [153], [154], [155], [156], [157], [158], [159], [160], [161], [162], [163], [164], [165], [166], [167], [168], [169], [170], [171], [172], [173], [174], [175], [176], [177], [178], [179], [180], [182], [183], [184], [185]). Among those not using BMI, weight, and body composition [137, 143], and WC [144] were reported. Results were statistically significant for the main outcome, i.e. related to obesity measures, in most (n = 50) articles. Change (n = 43) and associations (n = 23) were the most common analyses. One article reported prevalence [151]. PA behavior or mental health status were not reported as a health outcome, but were part of the intervention.

A recurrent theme across many studies was combining dietary changes, PA, and behavioral support, which consistently improved body composition, metabolic health markers, and quality of life [177]. For example, interventions incorporating elements such as the MD (measured by the MD index), resistance and aerobic exercise (measured by muscle strength test and lactate threshold), and stress management effectively reduced obesity-related (bio)markers and enhanced psychosocial well-being and emotional management [136, 137, 176]. Furthermore, integrating education and structured guidance, whether in nurse-led programs or community-based settings, has proven effective in fostering long-term healthy behaviors, even amidst varied demographic challenges such as those presented by MetS or obesity in adolescents [112, 124, 142]. Although some interventions focused specifically on nutrition or PA, the most successful outcomes often came from those addressing psychological aspects, such as stress management to reduce perceived stress and coping strategies, highlighting the holistic nature required for tackling obesity [155, 185]. However, psychological aspects, besides being part of the intervention, were not reported as outcome endpoints in the studies.

The conclusions from these studies emphasize the effectiveness of comprehensive, multimodal lifestyle interventions toward achieving significant health outcomes, particularly with respect to weight loss and metabolic improvements. Ultimately, these findings highlight the importance of a personalized and sustained lifestyle modification approach in effectively improving physical and mental health parameters across diverse populations.

#### Limitations

Some studies relied on self-reported data for dietary intake, PA, and other lifestyle behaviors, which introduced the risk of recall bias and inaccuracies, affecting the validity of the findings [124, 157, 170, 182]. For mental health, though questionnaires were typically employed to assess mental status, additional, more objective tests and even imaging have been called for [187]. Furthermore, mental status was never reported as an outcome endpoint. These limitations highlight the need for more rigorous study designs, including larger and more diverse sample populations, the use of objective measures, and comprehensive follow-up periods, to enhance the reliability and applicability of lifestyle interventions aimed at managing obesity, mental health, and related health conditions.

Furthermore, only 1 study utilized the metabolic (bio)markers [100] in combination with other markers including low-calorie diet, health, and PA promotion and stress-management intervention. This highlights the need to combine intervention, behavioral markers, and biomarkers to enhance the effectiveness of obesity prevention strategies. Additionally, the combination of the markers used was highly variable, making it challenging to synthesize the evidence. More studies are needed that systematically capture and test different markers to address this issue.

### Group 3: diet, PA, and any other component

#### Overview of articles retrieved, type of studies, number of combinations, and main findings

This group included 48 articles combining either diet, PA, and traditional markers (n = 26); diet, PA, and omics (n = 8); diet, PA, and genetics (n = 6); or diet (exceptionally allocated within this group) and traditional markers (n = 8). Most studies took place in Europe (n = 16) and the United States (n = 15). The sample size in this subgroup ranged from 11 to 11,281 participants, with a mean of 476 participants. The majority was conducted on adults only (n = 25, 52%), adults and older adults (n = 8, 17%), and children (n = 6, 13%). Most studies (n = 34, 71%) presented findings for both sexes, whereas 9 studies focused exclusively on females (19%). Most of the articles were multiple-arm intervention studies (n = 32, 67%); 3 of them were nonrandomized. Additionally, there were 8 pre–post (single-group) intervention studies (17%), 4 cross-sectional studies (8%), 2 prospective studies (4%), and 2 pilot studies (4%). The duration varied from 14 d [188] to 4 y [189].

#### Discussion on studies combining diet, PA, and any other components, and measured markers

The interventions generally focused on assessing the effects of combining global PA promotion, PA recommendations (e.g. walking or jogging 1 h/wk, 3 times per week), specific exercise programs (including mainly aerobic exercise and strength training), diet based on guidelines, eating behavior and education, general healthy diet promotion, specific diet (mainly low/hypocaloric diet) or diet supplementation [e.g. curcumin and anthocyanin, fiber-containing dietary, seaweed (poly)phenol], and behavioral change support (e.g. individual and group meetings, tele-health consisting in virtual nutrition sessions) to encourage weight loss.

All in all, of the 48 articles the most frequent interventions and (bio)markers investigated were “diet based on guidelines” (*n* = 15), “low-calorie diet” (*n* = 14), “aerobic PA” (*n* = 9), “PA promotion” (*n* = 9), “vigorous PA” (*n* = 7), “light to moderate PA” (*n* = 6), “dietary indices” (*n* = 5), and “eating behavior and education” (*n* = 5).

Measurement methods for PA behavior assessment included accelerometers (n = 9 [188, [190], [191], [192], [193], [194], [195], [196], [197]]), questionnaires (n = 6 [94, 119, 121, 192, 198, 199]), pedometers (n = 2 [200, 201]), activity trackers (Fitbit, n = 1 [202]), and double-labeled water (n = 1 [203]). However, only 10 studies (21%) included variables related to the PA behavior exposure in their analysis (i.e., daily steps, energy expenditure, moderate-to-vigorous PA, sedentary time, and sleep score). Diet was assessed in 20 studies using a 3- to 4-d food diary (n = 13 [119, 121, 190, 191, 198, 199, 201, [204], [205], [206], [207], [208], [209]]), 24-h dietary recalls (n = 5 [188, 192, 195, 196, 210]), 14-d electronic food diary (n = 1, [188] and FFQ (n = 1 [114]).

All studies included anthropometrics as a method for measuring the outcome (obesity), with anthropometrics being the only method used in 30 studies and BMI being the only method used in 10 studies. Other methods used for obesity measurement were BIA (n = 9), DEXA (n = 8), MRI (n = 3), and CT scan (n = 1).

In summary, only a few number of studies looked to multimodal biomarkers without considering diet and PA, considering, instead aspects such as genetics or mental aspects combined with blood traditional obesity markers.

#### Limitations

Most of the intervention studies (n = 40, multiple-arms, and pre–post single group intervention studies combined) did not assess PA behavior or dietary patterns in detail and focused on the effect of the intervention on the outcome (which was not always obesity). Consequently, no relationship between the PA behavior or dietary measurements and obesity was directly assessed in most of the studies. Thus, only associations between the study group (or time in pre–post single-group intervention studies) and outcomes were tested. In 4 out of 5 studies of this group, correlations/associations between biomarkers and obesity were only tested in about half of the studies of this group (n = 25) (Supplemental Figure 1, case B).

### Group 4: diet, -omics, and any other component

#### Overview of articles retrieved, type of studies, number of combinations, and main findings

Out of 43 articles associating diet, omics, and other components, 24 dealt with diet and omics (subgroup 1), 3 with diet, genetics, and omics (subgroup 2), and 16 with diet, omics, and traditional markers (subgroup 3; Supplemental Table 3).

In subgroup 1, of 24 articles, most studies were conducted in the United States [129, [211], [212], [213], [214]] and Europe [[215], [216], [217], [218], [219], [220], [221], [222], [223], [224], [225], [226], [227]]. A majority were RCTs (n = 18) [129, [211], [212], [213], [215], [216], [217], [219], [220], [221], 223, [226], [227], [228], [229], [230], [231], [232]]. They were completed by 2 prospective cohorts [214, 233], 2 observational studies [218, 225], 1 pilot RCT [222], and 1 nonrandomized intervention [224]. The sample size in this subgroup ranged from 20 to 726 participants, with a mean of 134 participants. The studied populations were mainly adults (n = 17) [[211], [212], [213], [214], [215], [216], 219, 220, 222, [224], [225], [226], [227], 230, 231, 233], but older people [129], adults and elderlies [218, 221, 229], children [228], children and adults [232], and infants [217] were also studied. Durations for interventions varied from 3 wk to 1 y.

Subgroup 2 (diet, genetics, and omics) included mostly studies from United States [234] and Europe [235, 236]. The group was composed of 1 RCT [234] pilot RCTs [235, 236]. The sample sizes in these 3 studies were 24 [236, 62 [236], 62 [235], and 1976 [234]. Two studies investigated adults [235, 236], whereas the other study examined both adults and older adults [234]. The duration varied from 1 menstrual cycle to 26 wk.

Finally, subgroup 3 had a total of 16 studies, most were conducted in the United States [237, 238] and Europe [[239], [240], [241], [242], [243], [244], [245]]. Ten of which were RCTs [128, [238], [239], [240], [241], 244, [246], [247], [248], [249]), four were cross-sectional studies [237, 243, 245, 250], and two were non-randomized interventions [242, 251]. The sample size ranged from 23 to 286 participants, with a mean of 113 participants. As for the 2 previous subgroups, adults were the main studied population (n = 12) [128, [239], [240], [241], [242], [243], [245], [246], [247], [248], [249], 251]. Two studies investigated adults and older adults [238, 244]: 1 studied children [250], and one studied infants and adults [237]. The duration of interventions varied from 3 d to 50 wk.

#### Discussion on studies combining diet, omics, and any other components, and measured markers

When the diet and omics subgroup 1, several studies have shown that omics can be an efficient approach to predicting which diet is most appropriate for each individual. One of the most frequently assessed exposure measures in this subgroup was gut microbiota composition (n = 16/24 studies). Some of the studies suggested that baseline gut microbiota profile, e.g. Prevotella-to-Bacteroides ratio (P/B, assessed, e.g. by 16S rRNA or metagenomics), could influence the efficiency of a certain diet or nutrient intake [219, 220, 226, 227, 233] with respect to the outcome (weight-related change). Typically, individuals with a high baseline P/B ratio showed an increased body weight loss when consuming fiber-rich whole grain diets compared with lower-fiber, refined wheat diets, whereas this difference was not observed in individuals with a low P/B ratio [220, 226]. It is well-accepted that fiber-degrading bacteria could contribute to elevated levels of anti-inflammatory short-chain fatty acids (SCFA), which may be beneficial against weight gain and related metabolic changes [252]. Other factors, including transcriptomic, DNA methylation levels, and metabolomic biomarkers, were shown to be potential biomarkers of body weight regulation, BMI reduction, or changes in fat and visceral adipose tissue mass under specific diets [129, 215, 222]. Other studies in subgroup 1 showed that diet (again with a focus on fiber intake), nutrient intake (e.g. low protein intake, phytochemical intake), and prebiotics could affect the gut microbiota composition and metabolic profile, which could impact body weight changes and metabolic risk factors [212, 213, 216, 217, 221, 223, 225, 228, 229]. In subgroup 1, the primary outcomes assessed were changes in BMI, WC, body composition, and body weight. They were mainly measured by anthropometry (n = 12) [214, 216, 217, 219, 221, 224, 227, 229, [231], [232], [233]] and, in some studies, also by DEXA (n = 8) [129, 211, 212, 218, 222, 225, 226, 228] or BIA measurement [230]. Of the 24 studies, 19 reported ≥1 obesity-related outcome that was significant (Supplemental Table 3), of which 9 were associations, 7 were correlations, and 10 were changes.

In subgroup 2, genetic components were also considered in addition to diet and omics. Genetics can also influence the response to dietary changes, a field known as nutrigenetics. For instance, one study showed that a low copy number of the salivary amylase gene (AMY1)—a biological predictor of postprandial glucose control and weight status—affected weight loss depending on the baseline P/B ratio (assessed by 16S rRNA) when following a New Nordic Diet—high in fiber, whole grain, intrinsic sugars, and starch—or an average Danish (Western) Diet [235]. On the other hand, both gene expressions, as detected by transcriptomics of several breast cancer biomarkers and metabolic markers, were shown to be impacted by diet, the former being influenced by a continuous energy restriction, whereas fluctuations in the concentration of several urine and serum metabolites, along with marked weight loss, were associated with intermittent energy restriction, an example of chrononutrition and low-calorie intake. The latter study corresponded to 2 consecutive days of 65% energy restriction and an MD that met the energy requirements for the remaining days of the week [236]. In another study, the 3 components of diet, omics, and genetics were integrated into a machine-learning approach, and obesity status was successfully predicted, further supporting the use of genetics in conjunction with diet and omics for obesity prevention [234]. Similar to subgroup 1, BMI, WC, and body composition were the assessed outcomes measured through anthropometrics [234] combined with DEXA [235] or BIA [236]. All 3 studies in this subgroup reported significant findings (Table 3); 2 reported changes, 1 an association, and 1 a correlation.

Finally, in subgroup 3, traditional markers were considered in addition to diet and omics. The studies demonstrated that dietary patterns can influence various metabolites, including fatty acid metabolism, carbohydrate metabolism, and inflammation and oxidative stress, which in turn impact weight loss [244, 245, 251]. One article demonstrated that consuming a high-rye fiber product within a hypocaloric diet resulted in modifications of the gut microbiota composition (assessed by 16S rRNA), associated with a reduced plasma C-reactive protein (CRP) concentration and an increased plasma butyrate concentration. These modifications led to increased weight loss [244]. On the other hand, the response to a diet can be influenced by factors such as adiposity, alongside gut microbiota species diversity and richness (assessed by gut microbiota metagenomics) [243]. Thus, taking into account adiposity status—whether the patient is overweight or has obesity—in association with gut microbiota composition could be a strategy to predict the effectiveness of a prescribed diet. However, although gut microbiota could be a biomarker of obesity, it cannot always translate the effects of a diet. For example, studies have demonstrated modifications in metabolic biomarkers after weight loss or changes in the plasma lipidome after the intake of orange juice, without altering the gut microbiota [240, 241, 248]. Again, BMI, body composition, and WC were the main outcomes measured by anthropometrics in 9 studies [128, 237, 238, 240, 242, 243, 248, 250, 251], combined with DEXA [244, 245], BIA [246, 247], MRI [241], or both MRI and BIA [249], and by DEXA only in 1 study [239]. Of 16, 13 studies in this subgroup showed significant findings (Supplemental Table 3), with 11 associations, 2 correlations, and 4 changes.

In conclusion, many studies have shown significant changes, associations, and correlations between obesity and a combination of dietary, omics, genetics, and traditional biomarkers, highlighting a potential added value in integrating these components together to better assess the risk of obesity of individuals and provide them personalized dietary adjustments.

#### Limitations

Overall, associating omic components with diet, genetics, or traditional marker measurements could be an efficient strategy to better understand the implications behind obesity and how to prevent it. However, there are limitations, such as the frequent use of self-reported food questionnaires, which introduce a non-negligible bias to the data, especially when estimating fiber and fiber-type intake in such studies. Additionally, partly related to analytical costs, the sample size was often small, and the lack of controls, such as placebo or nonintervention controls, further limited interpretations in this domain. Finally, the number of observational studies was relatively low, as were studies that entail omics tools in clinical settings. Thus, omics tools have not yet been integrated into general clinical practice.

### Group 5: diet, genetics, and traditional markers

#### Overview of articles retrieved, type of studies, number of combinations, and main findings

A total of 24 articles were included in this group, exploring the relationship between diet as well as genetics, including epigenetic markers (n = 16) as well as diet, genetic/epigenetic, and traditional markers (n = 8), and obesity risk. Eighteen of the articles were from Europe [150, 215, 222, 225, [253], [254], [255], [256], [257], [258], [259], [260], [261], [262], [263], [264], [265], [266]], 6 from North America [234, [267], [268], [269], [270], [271]], and the remaining 5 from Asia and the Middle East. Regarding sex, 5 articles were only focused only on females [150, 262, 264, 269, 272], only 1 study on males [263], and all others were done on both sexes. Regarding combinations of diet and genetics (n = 13), other components also included traditional markers (n = 11). The total sample of individuals was 48,088 from different population groups, although the cohort sizes varied from 44 to 13,692 individuals (6846 mother–child pairs [273]). These studies, except for 2 [268, 273], were focused on adults.

The most common analyses included group comparisons, regression models, and correlation analyses, with authors aiming to examine relationships, associations, and differences among variables while accounting for potential confounders and multiple testing. Studies encompassed various methodological approaches, including GWAS, RCTs, Mendelian randomization analyses, and cross-sectional studies. Although the methodologies used varied, RCTs (18/29) [150, 215, [253], [254], [255], [257], [258], [259], [260], [262], [263], [264], [265], 267, 270, 271, 274, 275] and observational studies [225, 261, 266, 268, 272, 273, 276] (7/29) were predominant, in addition to 2 intervention studies [222, 256], and 2 prospective cohort studies [234, 269].

#### Discussion on studies combining diet, genetics, and any other components, and measured markers

Genetic variants were assessed mainly through PCR methods [253, 254, [258], [259], [260], [261], [262], [263], [264], [265], 267, 268, [270], [271], [272], 274] or arrays [256, 269]; only 1 study used whole-genome sequencing data [275], possibly due to the high price tag still associated with this analysis. DNA methylation was assessed using Illumina EPICS arrays [215, 222, 225, 234, 257]. Regarding dietary endpoints, studies focused on specific dietary patterns and components such as phytochemical intake [253, 263], vitamins and minerals [254], adherence to dietary guidelines [254], caloric restriction and low-calorie diets [258, 259, 262, 265, 267, 270, 271], macronutrient-specific approaches including low-fat [259, 260], low-carbohydrate [265], and protein-rich [265], as well as dietary indices [258, 274] and food group–based intakes such as vegetables [255], dairy products [275], and probiotics [150]. Other dietary endpoints included supplementation practices [255, 256, 261], reflecting a broad spectrum of nutritional exposures assessed across the studies.

Integrating genetic and epigenetic markers as biomarkers for obesity risk offers promising avenues for personalized nutrition and targeted interventions. The findings indicated that genetic predisposition alone is not deterministic; rather, it interacts with lifestyle, diet, and other environmental factors to modulate obesity risk. For example, individuals with FTO risk alleles exhibited different responses to caloric restriction and macronutrient composition [276], whereas those with APO B polymorphisms demonstrated heightened inflammatory responses, exacerbating metabolic complications [276].

Additionally, epigenetic modifications play a crucial role in obesity by altering gene expression in response to dietary patterns and metabolic states. DNA methylation changes in key metabolic genes may serve as early indicators of obesity risk and efficacy of interventions [262]. Epigenetic modifications, such as DNA methylation changes in metabolic genes, were identified as predictors of weight loss success, as demonstrated in a lifestyle intervention study [257]. Moreover, a predictive model based on DNA methylation patterns showed to improve weight loss outcomes by tailoring dietary interventions to individual epigenetic profiles [222]. Machine learning approaches that leverage genome-wide and epigenome-wide interactions improved obesity risk prediction by integrating genetic and lifestyle factors [234]. Regarding measurements related to obesity mostly included changes in body weight, WC, adiposity, and BMI by anthropometry.

Taken together, these studies highlight the heterogeneity of obesity-related genetic predisposition and the significant influence of dietary and environmental factors in modulating genetic risk. For example, FTO gene polymorphisms were associated with dietary adherence to a 2-y caloric restriction intervention and weight loss outcomes [261]; PPM1K genetic variants impacted insulin resistance and β-cell function during weight loss [259]; and vitamin D receptor polymorphisms were associated with decreased obesity markers during weight-loss intervention and vitamin D supplementation in persons with overweight/obesity [254]. In subgroup 2, which included traditional markers, there were studies that suggested a role for genetics/epigenetics in the underlying inflammatory processes. APO B gene deletion allele was associated with inflammatory markers in persons with diabetes with obesity [276], whereas nutrigenetic interventions, such as polyphenol intake, had clear effects on lipid metabolism and inflammation [253, 267], and TCF7L2 (a transcription factor important for adipocyte metabolic regulation) polymorphisms affected inflammatory responses to legume-based diets [274]. Similar to the latter study, clear gene-diet interactions were observed for milk consumption and obesity risk [275]. Furthermore, the modified Nordic-style diet score was inversely associated with visceral fat levels and cardiovascular risk factors [272], whereas polymorphisms in the dopamine receptor may represent a marker for children with poor behavioral responses to unhealthy local food environments [268]. There was also an association between paraoxonase-1 gene (responsible for a group of antioxidant enzymes participating in organophosphate hydrolysis) methylation and antioxidant intake in patients with MetS following an energy-restricted diet [225].

#### Limitations

Genetic and epigenetic components serve as valuable biomarkers for assessing obesity risk and tailoring dietary and therapeutic interventions. The interplay between genetic susceptibility and environmental factors highlights the importance of personalized approaches in managing obesity. However, their use is still far from clinical practice, in part due to cost-related aspects and partly due to the more sophisticated technology involved, as well as the need for skilled personnel to interpret data. Future research should focus on integrating multiomic approaches, combining genomics, transcriptomics, metabolomics, and microbiome data to refine risk prediction. Furthermore, large-scale longitudinal studies and intervention trials are necessary to validate the efficacy of genetic and epigenetic markers in clinical and public health applications.

### Group 6: diet, mental aspects, and any other component

#### Overview of articles retrieved, type of studies, number of combinations, and main findings

A total of 18 articles were included in this group, addressing various psychological and/or dietary aspects, including weight loss and weight control strategies, as well as improvements in mental health status. Most articles were from Europe [115, 127, [277], [278], [279], [280], [281], [282]] and North America [[283], [284], [285], [286], [287], [288], [289], [290]], 1 from Brazil [291], and 1 from Egypt [292]. The total sample of individuals was 5115 from different population groups, but individual studies ranged from 7 to 75 participants. The length of the interventions varied from 4 wk to 8 y. The studies assessed the general elderly population [282], older adults with prostate cancer [285], children and adolescents [283, 291], adults [115, 127, [277], [278], [279], [280], [281], 288, 290], adult females with or without pre-/postmenopause [284, 286, 287, 292], and military personnel [289]. The designs of these studies were heterogeneous, although RCTs [279, 280, [287], [288], [289], [290]] and observational studies [278, 281, 283, 284] predominated, in addition to 3 intervention studies [282, 285, 292], 3 RCTs [115, 127, 286], 1 cross-sectional [291], and 1 cohort study [277].

The most common statistical analyses were adjusted mean comparisons (t-student/ANOVA/ANCOVA (analysis of co-variance)) under the umbrella of the general linear model. Median comparisons were carried out for nonparametric samples. Regression and correlation analyses were also performed, ranging from correlations to associations and changes, which allowed the authors to explore relationships, patterns, and trends among variables.

#### Discussion on studies combining diet, genetics, and any other components, and measured markers

Of 18 reports, 10 studies focused on the components of diet and mental health [127, 279, [282], [283], [284], [285], 288, [290], [291], [292], 7], 7 studies included additional traditional markers [277, 278, 280, 281, 286, 287, 289], and 1 study also examined genetic aspects [115]. Dietary aspects typically involved dietary interventions with or without counseling, and questionnaires were used to assess dietary habits. Psychological aspects were evaluated using questionnaires or scales that measured mood, stress, anxiety, depression, body image, self-efficacy, quality of life, cognitive activities, and, in some cases, electroencephalograms. General traditional markers included the determination of serum cytokines and growth factors, calcaneal BMD, cortisol concentration, and neuroimaging. The genetic studies included informing participants about their genetic risk [[293], [294], [295]]. As for the main outcomes, measurements and changes in body weight, WC, adiposity, and BMI were carried out by anthropometry, and in some cases, by bioimpedance or DEXA [[296], [297], [298], [299]].

The findings demonstrate a highly heterogeneous association between diet, mental health, and obesity, both in terms of trial design and population characteristics, as well as interventions and duration. These included dietary interventions that combined conscious control, counseling, or cognitive-behavioral therapy (CBT) and appeared more effective than dieting alone. In overweight females, short-term weight loss [284] and better-sustained weight loss were observed with early behavioral control [286]. In premenopausal females, restrictive diets with conscious control did not negatively affect their eating behavior and perceived stress [287]. Among older adults, a study found that a more intensive and personalized intervention, including medical consultations, PA, and nutrition education, was more effective for weight loss and improvement of physical indicators than simply providing written recommendations from the medical doctor (control group). In terms of mental health, only depression improved (in both groups), but not anxiety [282].

Furthermore, it was demonstrated that overly rigid weight management strategies can be detrimental, underscoring the importance of a flexible approach to dieting for achieving and maintaining sustainable weight control [127]. For older adults with prostate cancer, healthy dietary counseling and stress reduction techniques decreased body weight and adiposity, as well as reduced prostate-specific antigen and increased sex hormone-binding globulin [285]. Another study, although no significant results were obtained, suggested that overweight/obese individuals have a mismatch in their eating habits relative to their natural chronotype, which could be affecting their weight. In contrast, individuals with normal weight align their eating patterns with their biological rhythms. This highlights the importance of designing diets tailored to the chronotype [278], i.e. chrononutrition. Another study emphasized the value of including genetic information in consultations for individuals with obesity. This approach showed to improve long-term mood in individuals with a family history of obesity compared with the group that did not receive this genetic information [235].

Other strategies include CBT or similar interventions. One study in the adult population showed that a CBT-based intervention helped people with overweight/obesity to lose weight and improve cognitive control. They demonstrated greater sustained attention, inhibitory control, and performance monitoring compared with the control group (who received written guidelines only). This supports using CBT to improve both physical health and mental self-control [288]. Nutrition education with realistic goals and lifestyle information seems to be another strategy to achieve health and mood benefits, especially in individuals with obesity and those with a genetic predisposition [115, 289]. Another study (though with nonsignificant results) suggested that inhibitory control training can reduce food cravings [290]. Another strategy would be to consume a specific food product to improve certain aspects of mental health. A study in postmenopausal females proposed that the intake of products rich in unsaturated fatty acids improved symptoms of anxiety and depression compared with phytoestrogen supplementation [292]. A pilot study validated controlled trials of probiotics for treating depression in MetS, highlighting the need to investigate their relationship with inflammatory markers [280]. Alterations in inflammatory markers were also documented in patients with anorexia nervosa and binge eating disorder [281].

In patients with type II diabetes, low lactate concentration was linked to increased body weight and increased postprandial hunger. Low fasting lactate may indicate an impairment in the brain’s ability to utilize alternative energy sources, such as lactate, in place of glycogen, thereby affecting the central regulation of energy balance regulation. This deficit could generate a signal of energy shortage, leading to increased hunger after meals [277]. Adiposity in middle age was found to affect executive functioning negatively, but not global cognitive function [279].

Thus, the main conclusion of this section is that dietary interventions, in conjunction with CBTs or behavioral strategies, delivered through face-to-face sessions and visits, or monitored via continuous Apps, produce better results with respect to weight loss or related markers than dietary intervention alone.

#### Limitations

Limitations in thus group include especially a limited number of studies, with generally only a few participants, which limits the generalizability of the findings, also in light of the heterogeneity in study populations.

### Group 7: PA without dietary, but any other component

#### Overview of articles retrieved, type of studies, number of combinations, and main findings

We identified 16 studies addressing multicomponent biomarkers based on PA behavior (excluding diet) in relation to overweight and obesity (Table 3). Most studies included PA and mental health (n = 7), followed by PA and traditional markers (n = 4). Most studies originated from North America (n = 7) [118, [300], [301], [302], [303], [304], [305]] and Europe [52, [306], [307], [308], [309], [310]]. They primarily focused on adults, with 3 also including older adults [118, 303, 311], 1 solely on older adults [306], and 3 on adolescents [300, 309, 312]. A systematic review [52] analyzed 12 studies on active working-age populations. Participants (ages 12–79 y) included healthy individuals [302, 303, 305, 309, 312], those with overweight or obesity [52, 118, 300, 301, 305, 307, 308, 311, 313], with type 2 diabetes [306], or coronary artery disease with obstructive sleep apnea [310]. Excluding the systematic review, the sample size had a mean of 2189 (range: 20–27,158). The majority of studies were RCTs lasting between 8 wk and 24 mo [118, 300, 301, 306, 307, 311, 313] or cross-sectional studies [302, 303, [308], [309], [310], 312]. Additionally, we found 1 secondary data analysis of a clinical trial [304], 1 longitudinal population-based cohort [305], and 1 systematic review with meta-analysis [52]. The most common analyses were correlations, associations, and changes, with authors aiming to explore the relationships, patterns, and trends among variables.

#### Discussion on studies combining PA and any other component except diet and measured markers

Movement, including PA and sleep, was assessed using both objective (e.g. accelerometer, polysomnography, and sleep recorder system) [304, 305, [307], [308], [309], [310]] and subjective methods (e.g. 7-d PA Recall Questionnaire, Past Year Total PA Questionnaire, PA Rating for Children and Youth) [118, 302, 303, 312]. Additionally, several studies incorporated structured exercise programs [300, 301, 306, 311, 313].

Most studies objectively collected outcome measures using methods such as anthropometrics, DEXA, or BIA. However, 2 studies relied on self-reported anthropometric measures [118, 302]. BMI was the primary outcome in all the studies, but 3 studies also analyzed it in combination with WC [300, 306, 310], whereas 5 studies complemented BMI and WC with body composition metrics such as lean and fat mass [301, 303, 307, 309, 311].

The studies highlight the complex interplay between PA, other components of 24-h movement such as sleep, and obesity, along with other influencing variables such as genetics, traditional biomarkers, and mental health. Several of these studies have emphasized the beneficial effects of PA on obesity-related outcomes in conjunction with metabolic and inflammatory markers [300, 301, 306, 313]. For instance, biomarker analyses revealed that reductions in adipose tissue through exercise affect systemic metabolites, lower proinflammatory cytokines (IL-6, TNF-α), and improve the balance between pro- and anti-inflammatory markers—a hallmark of manifest obesity—further offering insights into cardiometabolic improvements [301, 311]. Sleep duration also plays a critical role, as shorter sleep duration was linked to higher BMI, weight gain, and increased emotional stress [305, 308], also proposing sleep as a considerable risk factor related to emotional well-being and overall energy homeostasis. Not too surprisingly, psychological outcomes, such as body satisfaction/esteem in boys, improved with PA, which was mediated through BMI [312], suggesting that psychological and emotional aspects, potentially linked to neuroendocrine or stress-related biomarkers, may play a role in obesity risk assessment and prediction. However, long sleep duration may not reduce BMI in all cases. Genetics may modulate the PA-obesity relationship, as observed, e.g. with UCP1 polymorphism (related to body heat production) and its association with WHR in adolescents [309], also proposing a more personalized approach including genetic aspects.

#### Limitations and perspectives

Although PA and multimodal approaches were effective [52,307], outcomes were specifically influenced by factors such as baseline health conditions, genetic predispositions, and sleep quality. This underscores the need to target integrated interventions to address obesity and its comorbidities comprehensively.

### Group 8: combinations without diet or PA

#### Overview of articles retrieved, type of studies, number of combinations, and main findings

Of the retained articles, 7 assessed multidomain biomarkers without considering diet and PA [[314], [315], [316], [317], [318], [319], [320]], with the majority considering genetics and traditional markers (n = 4) [[317], [318], [319], [320]], followed by mental aspects and traditional markers (n = 2) [315, 318]. The studies report results obtained from different continents, namely Asia [314], North America [316, 318], Europe [317, 319], and Africa [320]. Three include 2 studies in children [317, 319], 3 in adults [314, 316, 318], and 3 in the older adults population [314, 315, 318]. Another study also included adults, specifically pregnant females [320]. The number of participants in the studies ranged from 56 to 378,877. Most studies were observational (n = 4), in which the association between anthropometry (BMI, body weight, and/or body fat mass) and various parameters, such as neurocognition, bone density, and blood parameters, was found to be statistically significant. Among the 3 intervention studies, the type and time of interventions varied, with 1 having a 6-mo exercise and nutritional intervention [315], whereas in the other 2, individuals received lifestyle recommendations for 12 wk [316] or 8 mo [319].

#### Discussion on studies combining PA and any other component except diet and measured markers

Regarding the assessment of genetics, molecular biology approaches were employed for DNA extraction and allele-specific polymerase chain reaction to identify polymorphisms in various genes. In the reviewed studies, traditional blood markers, including glucose, triglycerides, LDL, HDL, and total cholesterol, were assessed using conventional biochemical methods.

As for the main outcomes, BMI and body composition were the predominant measures. With respect to the main obesity outcome, Yuan et al. [314] reported, based on a Chinese population of adults (including older people) that BMI exerted interactions with BMD and vitamin D status. Though higher BMI may be related to lower vitamin D status, low BMI was associated with lower BMD (higher osteoporosis risk). In some studies, the measured outcomes were related to changes in blood parameters [316, 319] and cognitive function [315, 318]. This highlights that looking at a single biomarker for the main outcome may be insufficient, especially in populations including older adults, where factors such as osteoporosis and sarcopenic obesity could play a role.

The genetic background and the presence of specific SNPs appeared to be associated with BMI, for instance, through SNPs related to vitamin D bioavailability, which impacted bone mass in the long term [314]. Another genetic study [317], a cross-sectional study, conducted in children assessed the role of the proinflammatory cytokine IL-6 gene polymorphisms in energy expenditure and the development of obesity and/or metabolic diseases. The study concluded that different polymorphisms in the IL-6 gene may modulate its expression to determine nutritional status in both healthy-weight and children with obesity. In one study, variations in the first intron of FTO constituted a risk factor for early-onset obesity, although no impact on weight loss or serum levels of blood glucose, triglycerides, and cholesterol (LDL-c and HDL-c) was noted [319]. Although the exact role of this gene is not yet known, it has been proposed that it may play a role in the hypothalamic–pituitary–adrenal axis [321].

Regarding mental health and genetics, SNPs linked to higher body fat mass were associated with better visual memory. Although the effect of obesity on cognitive function is unlikely to be causal, an association between poor visual memory and lower adiposity has been reported [318]. However, it is important to highlight that this study did not find a relationship between obesity and visual memory, but rather suggests that the lower visual memory observed in middle age (50 y old) may be associated with mental diseases (e.g. Alzheimer’s disease), which can lead to body weight loss. This interaction between obesity and visual memory was also researched in another study [315], though without findings of a significant association between these variables. This study in older adults, however, found lower scores in executive function and verbal memory among individuals with higher BMI [315]. In these persons, a higher BMI was also associated with worse metabolic profiles (higher Homeostasis Model Assessment of Insulin Resistance (HOMA-IR), leptin, and IGF-1 levels) and greater CRP levels, suggesting that a combination of these traditional markers with cognitive aspects may have merit in predicting obesity.

One study also reported that gestational weight gain was predicted by obesity and age [320]. Overweight, more frequently observed in older females, was associated with increased weight gain during pregnancy. Moreover, parity was also a factor in weight gain during pregnancy; multiparous females had decreased odds of excessive weight gain compared with primiparous females [320]. Whether such factors have an impact on weight beyond pregnancy is unclear.

A final study focused on how to make interventions for obesity prevention more effective. The authors reported that multimodal interventions, namely lifestyle, exercise, attitudes, relationships, and nutrition, were more effective when reinforcement existed [316] compared with when absent. In adults, receiving prizes substantially enhanced short-term weight loss and reductions in weight, which were associated with positive changes in clinical biomarkers, such as decreases in total cholesterol and 24-h ambulatory heart rate.

In conclusion, the 7 different studies reinforced the notion that the multidimensional nature of obesity is also reflected in the consideration of various biomarkers, proposing the inclusion of traditional biomarkers, as well as genetics, and specifically different SNPs, to increase the predictability of the risk of developing obesity.

#### Limitations

In this category, the number of studies was very limited (n = 7), with also small study sizes, in some cases below 60 [315, 317, 320], and limited regarding the amount of study duration (e.g. 12 wk [316].

### Group 9: any 4 components combined

#### Overview of articles retrieved, type of studies, number of combinations, and main findings

We identified 43 studies addressing multimodal outcomes with ≥4 components. Most studies investigated a combination of 4 components, most commonly diet, PA, mental aspect, and traditional markers (n = 22). This was followed by diet, PA, omics, and traditional markers (n = 6), whereas a few studies included 5 components, such as diet, PA, genetics, omics, and traditional markers (n = 3).

Geographically, the studies originated from different countries, including especially Brazil [54,186, [322], [323], [324], [325]] and the United States [117, 120, [326], [327], [328], [329], [330], [331], [332]], among others. Two studies included several countries [333, 334]. The studies focused on adults [117, 120, 122, 123, 130, 324, [326], [327], [328], [329], [330], [331], [332], [334], [335], [336], [337], [338], [339], [340], [341], [342], [343], [344], [345], [346], [347], [348], [349], [350]], as well as children and/or adolescents [54, 186, 322, 323, 325, 333, [351], [352], [353], [354], [355], [356]]. Across the 43 studies, participant ages ranged from 2 to 80 y, including adult individuals who were breast cancer survivors [120, 331, 344], infertile [335], premenopausal [117], or postmenopausal females [326], or presenting mild cognitive impairment [324]. In all studies, except 3 [117, 333, 353], the individuals were overweight or obese. One study [347] included both lean and obese participants. The majority of the studies were RCTs lasting between 13 d and 3 y [54,117, 120, 122, 123, 130, 186, [322], [323], [324], [325], [326], [327], [328], [329], [330], [331], [332], [333], [334], [335], [336], [337], [338], [339], [340], [341], [342], [343], [344], [345], [348], [349], [350], [351], [352], 356, 357]. Additionally, there were 3 cross-sectional studies [[353], [354], [355]] and an experimental before-and-after study [346]. The most common analyses focused on changes, with authors aiming to explore the effects of the interventions.

#### Discussion on studies combining PA and any other component except diet and measured markers

The multimodal interventions mainly focused on different caloric restriction approaches, PA enhancement, cognitive behavioral stress management, cognitive training, or behavioral therapy. Due to the multimodal approach, a large variety of outcome measures within the areas of diet, PA, mental status, omics, genetics, and biomarkers were determined in the 43 studies. The outcome measures within the area of diet included, e.g. dietary intake [117, 122, 123, 130, 186, 322, [324], [325], [326], [327], [328], 332, [334], [335], [336], 340, 342, 343, 345, [348], [349], [350], 352, 353], eating behavior [330, 336], and appetite ratings or perceived satiety [327, 342]. The outcome measures within the area of PA were muscle strength [54, 117, 122, 123, 130, 324, 327, 334, 335, 340, 345, [348], [349], [350], 352, 353] [331, 332, 339, 347], sedentary behavior (including screen time [328, 350, 353], muscle mass, and aerobic and anaerobic exercise capacities [117, 326, 327, 331, 348, 349], among others. The outcome measures within omics included metagenomic/microbiome analysis [342, 343, [345], [346], [347]], untargeted metabolomics [339, 346], and transcriptomics [349, 356]. Within genetics, SNP analysis [122, 123, 334, 337, 338, 340, 355], gene enrichment analysis [357], and GRSs [122, 123, 338, 340, 355] were included in some studies. The outcome measures within the area of mental status included, among others, quality of life [342], emotional and behavioral dysfunction [328], body satisfaction [348], perceived stress [330, 336], anxiety symptoms [130, 330, 350], neuropsychological tests [326], and mood state [324], and mood state [327]. The exposure measures within the area of traditional biomarkers were concentrations of monoamines [130], ghrelin (hormone related to hunger [120, 342], adiponectin [122, 323, 340, 348, 352], bone turnover markers [323], vitamin D status [325], and other appetite hormone levels [117, 340, 342], as well as cardiometabolic biomarkers related to carbohydrate and lipid metabolism, immune, and inflammatory biomarkers [54, 117, 130, 325, 326, [328], [329], [330], [333], [334], [335], [336], [337], 340, 342, 343, 347, 348, 350, 351, 357].

With respect to obesity, body weight and/or BMI were the primary outcomes in most studies, and changes in weight, WHR, WC, body composition, and body fat were often measured. Studies reported significant primary outcome measures related to changes in body weight, BMI, WC, WHR, or body composition [54,334, 336, 356]. For instance, the intervention (based on diet, PA, and group support) in the study by Walker et al. [334] led to significant differences between the control and intervention groups. The authors reported significant weight loss and decreased traditional markers of HOMA-IR, HOMA-B, and glucose in adults. In another study, the intervention for adults was based on cognitive-behavioral stress management [336]. In that study, the authors found a statistically significant decrease in cortisol, perceived stress, eating behavior, and a number of traditional markers, including fasting glucose, LDL-c, triglycerides, and HbA1c, in addition to reduced primary outcomes, weight, BMI, WHR, body and trunk fat [336]. A multimodal intervention (physical exercise, nutritional education, and psychological intervention) found improved body composition (WHR and body fat percentage) and cardiorespiratory fitness, but no effects on insulin biomarkers (glucose, insulin, and HOMA-IR) among adolescents aged 10 to 17 y [54]. One study intervening with nutritional education and PA found that miR-221-3p expression was positively correlated with the primary outcomes body weight, BMI, and WC, and negatively correlated with Quantitative Insulin Sensitivity Check Index in 7 to 16-y old girls [356].

#### Limitations

The remaining nonmentioned studies did not include a control group, whereas others had small sample sizes, focused on effects within groups rather than between groups, or did not report significant effects related to weight change.

## Overall discussion, strengths and limitations, and perspectives

### Overall discussion

When focusing on multimodal aspects related to the risk of developing obesity, the most common combinations have been, by far, diet and PA (Table 3). These have typically assessed adherence to general dietary guidelines and calorie reduction regarding diet and general as well as light to moderate PA promotion. Both diet and PA aspects are typically evaluated employing questionnaires, which is a shortcoming due to the high risk of bias. However, such combinations can be easily applied to a clinical context. In addition, many of these studies have neglected socioeconomic status [358], emotional aspects [359], or other aspects of total environmental exposure. Such exposure could be attempted to be captured by, e.g. the use of omics tools [359], also considering host factors such as genetic ones [360], all of which have been highlighted as potentially playing a role in the development of obesity. However, few studies have included or combined markers related to PA and diet with omics and genetic data, in part due to technical costs and hurdles related to their measurement, evaluation, and interpretation of the often complex data structure. Studies focusing on diet and PA have rather often included traditional and clinically measurable markers, such as blood sugar or lipid levels, to add some predictability with respect to obesity risk, though surely these markers also lack specificity, and it is arguable whether they precede obesity or are a consequence. A number of studies have also included mental health aspects, mostly focusing on lifestyle behavior assessment followed by perceived stress and cognitive/personality traits, due to the realization of the tight link between emotional well-being and the risk of developing obesity, perhaps especially during early life stages [361]. Specifically, stress was shown in some studies to foster the intake and even craving of energy-dense food items [362] as well as producing irregular eating patterns [363], which may result in weight gain. Thus, CBTs and behavioral strategies have emerged as effective approaches to address this relationship.

A challenge also appears in the integration of various markers into a combined analysis. Most studies did not attempt to evaluate the collected markers together; instead, they used basic statistical tools (mean effect size, mean difference, etc.) for intervention studies or association/correlation approaches, while considering some confounders to assess the relationship between individual markers and the obesity outcome. Few studies attempted to create an integrative score. The 2 most prominent methods for integrating findings from multimodal studies are either by summing up/weighing elements of a combined score [114, 128] or by algorithms creating a new combined score [364]. This lack of integration is also reflected in the absence of studies that employed machine learning [234, 365] or even artificial intelligence (AI) tools [366] in their interpretation. Such tools are much needed to foster an integrative examination of the combinations and personalized approaches of possible markers related to obesity [[366], [367], [368]]. For instance, a recent article [369] highlighted that a supervised learning algorithm, including, among other factors, age, sex, PA, selected dietary aspects, smoking, and alcohol consumption, was able to predict obesity rate with high accuracy (92%).

As the present review is not systematic, it is challenging to determine which combinations are the most prominent and have the best predictive value for the risk of obesity development. Judged by the proportion of articles with significant findings, this would involve combining ≥4 of the multimodal aspects considered in this article. However, this may be a crude indication, as it does not consider the quality of the obtained data or the statistical analyses involved. Nevertheless, combining individual risk factors into a combined score appears meaningful. Multimodal combinations have also been employed in the treatment of obesity [370], including bariatric surgery, drug administration, and dietary and mental counseling. However, practical limitations such as costs, the availability of instruments, and the interpretation of combined data remain challenging. Multimodal predictions have also been employed for further downstream analysis of obesity risk factors related to CVDs, revealing visceral adiposity, BMI, and triglycerides as good predictors. However, genetics, mental health, and omics were not included [371].

An interesting approach is likely to integrate individual components into a comprehensive index before combining various components into a single (bio-)marker. For dietary patterns, this would mean their combination into a dietary index, such as the alternative healthy eating index (AHEI) [47], diet quality index [43], or dietary antioxidant index [372], among others. For PA, this could involve employing a walkability index or a similar measure [373]. Regarding mental health, indices have been proposed for development [374]. Regarding traditional markers, possibly scores describing the risk of MetS or similar may be promising to integrate [375]. For genetics, GRSs such as the obesity risk score could be included [45], and for multiomics, integrative approaches have also been attempted with respect to obesity [376], as have been combining gut microbiota diversity and serum metabolomics for predicting childhood obesity risk [377]. However, to our knowledge, the creation of a “meta-index” combining indices across domains has not been attempted. A few studies in the present review have combined indices across different domains, e.g. the combination of Genome-Wide and Epigenome-Wide Gene–Gene and Gene–Diet (AHEI, dietary approaches to stop hypertension (DASH) diet score together with Mediterranean diet score (MDS)) interactions and changing key dietary factors individually, with 1.5% to 19.6% of subjects showing responses in changing obesity risk [234]; overall, ∼21.5% of individuals showed a response to ≥1 dietary change based on simulation.

Taken together, many novel approaches with multimodal character have been proposed, and despite their present limitations, they may offer advantages in predicting obesity onset and proposing early countermeasures. Identifying novel approaches and gaps will aid guiding research priorities and future directions in obesity prevention [54]. Such approaches can have clinical potential and public health implications, supporting personalized interventions, as well as policy development for obesity prevention and management [[378], [379], [380], [381], [382], [383]].

In this review, the overall findings underscore the value of incorporating diet, PA, and mental health into research protocols and showcase that the inclusion of additional markers from the area of omics and genetics is expected to be beneficial with respect to obesity prediction, though we could not quantify the added benefit further. The relatively high fraction of significant findings in combinations containing PA, diet, and mental health components (particularly groups 1 and 3) suggests that they represent a practical, feasible, and cost-friendly approach, balancing methodological rigor with practical constraints, such as study duration, resources, participant burden, and sophisticated equipment needed (Figure 2). The limited number of studies, including those involving omics and genetic endpoints, and the considerable heterogeneity of approaches perceived, may reflect the complex interactions between diet, genetics, and mental health, which may not always translate into measurable impacts within the context of the sampled studies, underscoring the need for careful consideration of these factors when designing impactful studies in the field of obesity research (Table 3).

### Strengths and limitations

A major subset of studies employed rigorous methodologies, with RCTs serving as the reference standard for evaluating causal relationships. Furthermore, a number of studies employed objective assessments, including accelerometry for PA measurement, biochemical markers for dietary intake, and DEXA for body composition analysis, improving the reliability of outcome measurements. Some studies included diverse study populations, covering multiple age groups, from children to older adults, incorporating both males and females. This diversity enhances the applicability of findings to broader populations and could allow for subgroup analyses that can identify potential sex- or age-specific associations. Additionally, several studies have employed multiomics approaches, integrating genomics, metabolomics, and gut microbiota profiling, which are emerging promising tools for personalized assessment of obesity risk. Several studies also considered lifestyle and psychosocial factors, including mental health and behavioral interventions, reflecting a holistic approach in the prevention of obesity. The integration of psychological aspects, such as stress, emotional well-being, and cognitive function, is a critical step toward understanding the multifaceted nature of obesity risk.

This scoping review itself has several strengths. First, it is the first comprehensive review that attempted to systematically map existing literature on multimodal (bio)markers for obesity risk, integrating biological, behavioral, and psychological components. Such a multidomain perspective provides a more holistic understanding of obesity risk factors compared with a focus on single biomarkers or interventions. By emphasizing the importance of multidomain obesity prediction models, this review supports the transition toward more personalized and evidence-based interventions for obesity prevention and management. Another strength is the systematic and transparent methodology employed in study selection, which follows PRISMA-ScR guidelines and adheres to a structured scoping review framework. Additionally, we applied a tiered approach to evaluate the quality of evidence, ensuring that only studies meeting specific methodological criteria were included in the quantitative mapping. By identifying knowledge gaps, such as the need for standardization in biomarker measurement and greater reliance on objective methodologies, we provide clear directions for future research and policies.

Some limitations must be acknowledged. One limitation is the exclusion of environmental factors, such as the built environment, from the present article, which is well known to be related to health outcomes [384], including obesity [385]. Another concern is the quality of the included studies, as methodologies varied widely in terms of study design, sample size, and duration. Indeed, several studies have cautioned that their results may not be representative of broader populations due to geographic, demographic, or socioeconomic constraints. Other common issues related to study design included the absence of control groups in several studies, hindering the drawing of causal conclusions, or not including relevant confounding factors such as SES, ethnicity, or pre-existing health conditions. Although RCTs were the most common study type, a significant number of observational and cross-sectional studies were included, which limits the ability to establish causality. Additionally, the duration of interventions was often brief, which hindered the evaluation of outcomes. Another problem was the huge heterogeneity in the (bio)markers studied and the lack of standardization. Variability existed in endpoint selection (e.g. specific inflammatory markers or –omics techniques), measurement timing (length of study and time of day), assessment tools (e.g. questionnaires, wearable devices, and blood measurements), and the varied populations (age, sex, and place of living). There was often limited use of objective (bio)markers due to constraints related to funding, time, and resources. Consequently, many studies relied on self-reported dietary intake and PA data, which are prone to recall bias and measurement inaccuracies. The use of more objective instruments—such as accelerometers for PA, metabolomics for dietary intake assessment, or biological markers of nutritional status (such as urinary sodium or 25-OH vitamin D in plasma), and high-throughput sequencing for microbiome analysis—remains underutilized despite their potential to provide more reliable insights into obesity risk factors. Regarding mental health, no study used imaging techniques to co-assess psychological aspects, which is perhaps a new avenue, relying exclusively on (validated) questionnaires. Such techniques have only recently been advocated as a complementary tool to validated questionnaires [386]. More behavioral therapies are also sought to promote changes in lifestyle and dietary habits, encouraging a healthier and more positive relationship to food and promoting sustainable, long-lasting changes [387], offering a holistic approach to tackle obesity and underlying factors.

Even for the main outcome, self-reported weight and height limited the value of the generated BMI, which by itself has been criticized for being a rather inaccurate measure of obesity [388, 389], for sure on the individual level [389, 390], rather than focusing on body fat distribution and related metabolic health [389]. Advanced imaging techniques, such as MRI and DEXA, were only applied in a subset of studies due to their cost and accessibility limitations. At least estimates of visceral fat derived from e.g. thigh circumference [103] should be included.

Furthermore, variations in measurement techniques, biomarker definition, and cut-off values across studies hindered direct comparisons and meta-analytical synthesis. Future research should prioritize standardized methodologies and increase the use of validated objective biomarkers to improve data accuracy and reproducibility. Increasing investments in funding and infrastructure for multi-omics research will be crucial for advancing this field.

Finally, regarding the combined evaluation of findings, despite the growing use of machine learning, AI, and advanced statistical modeling to analyze multimodal data, no studies included involved these tools, representing another important shortcoming that could otherwise facilitate the identification of complex interactions between biomarkers and obesity-related outcomes.

We also note that only a small proportion of studies explicitly reported the availability of open data or code. This limited transparency constrains reproducibility and precludes quantitative synthesis or meta-analysis, as also reflected by the absence of ML/AI applications.

## Perspectives

This scoping review highlights the crucial role of multimodal (bio)markers in enhancing the prediction and intervention strategies for obesity risk. By integrating biological, behavioral, and psychological factors, such approaches provide a more comprehensive understanding of obesity’s complex etiology compared with single-marker assessments. The increasing use of multi-omics technologies necessitates the further application of ML and AI tools, as well as objective measurements, to advance the field further. Challenges also remain regarding the standardization of biomarker selection, methodological consistency, and the long-term applicability of these approaches in clinical and public health settings.

Future research should prioritize large-scale, more long-term longitudinal studies that incorporate validated, objective, and reproducible biomarkers, thereby ensuring more precise risk stratification and effective, personalized strategies for assessing the durability of behavior changes and health benefits related to obesity [148]. Bridging the gap between emerging research innovations and practical implementation will be essential in translating these findings into impactful public health interventions. More use of technological advances, such as by validated smartwatches and their features [391] to determine PA behavior and health outcomes, should be employed. For nutrients such as minerals and vitamins, biomarkers of intake that can be measured in the bloodstream are desired. In addition, neuroimaging for mental health has also been generally underexplored. Even for the main outcome of obesity, more objective measures, such as visceral fat, as measured via BIA (bioimpedance), DEXA, or MRI, should be strived for, despite associated high costs. With respect to costs, few studies were designed to evaluate the cost-effectiveness of interventions and measures, which is paramount for understanding the potential for real-world application and scalability [167, 171], such as uptake in clinical environments.

## Author contributions

The authors’ responsibilities were as follows – FV, TB: designed the scoping review protocol, evaluated the articles, extracted data, analyzed the results, drafted the first version, and edited the final version of the manuscript; and AL-L, JTur, CB, YD, LM, SG, MDC, MR-M, JTurner, EL, MP-J, GR-H, RA, SF, RN, MGO, GGB, DR: were involved in designing the protocol, editing the final version, and writing relevant sections of the manuscript.

## Data availability

The data supporting the findings of this review are available on request, subject to approval by the HealthyW8 consortium.

## Funding

This research is being conducted as part of the HealthyW8 project, which received funding from the European Union’s Horizon Europe Research and Innovation Programme under Grant Agreement No. 101080645. JT, CB, SG, and MR-M are also funded by CIBEROBN (CB12/03/30038) of the Instituto de Salud Carlos III, Spain. YD has received funding from the EU Horizon 2020 project COVIRNA (grant agreement #101016072), the National Research Fund (FNR) (grants #C14/BM/8225223, C17/BM/11613033 and COVID-19/2020-1/14719577/miRCOVID), the COST Association (Actions #CA17129 and CA21153), the Ministry of Higher Education and Research, and the Heart Foundation-Daniel Wagner of Luxembourg. The FNR of Luxembourg supports MDC under the grant agreement Xpose no PRIDE23/18356118. DR and MP-J received funding from the Fundação para a Ciência e Tecnologia (FCT), Portugal (Grant 2020.03966.CEECIND and Grant 2023.06153.CEECIND), with MP-J also receiving funding from the EU MSCA-ERA Postdoctoral Fellowship ID Project: 101180615.

## Conflict of interest

The authors declare no conflict of interest.
