# Supplementary material for: Multimodal (Bio)Markers and Risk of Obesity – A Comprehensive Scoping Review
Source: Adv Nutr. 2025 Dec 24;17(2):100579. doi: 10.1016/j.advnut.2025.100579 (PMC12907118; doi:10.1016/j.advnut.2025.100579)
Supplement: multimedia component 2 [file mmc2.docx]

**
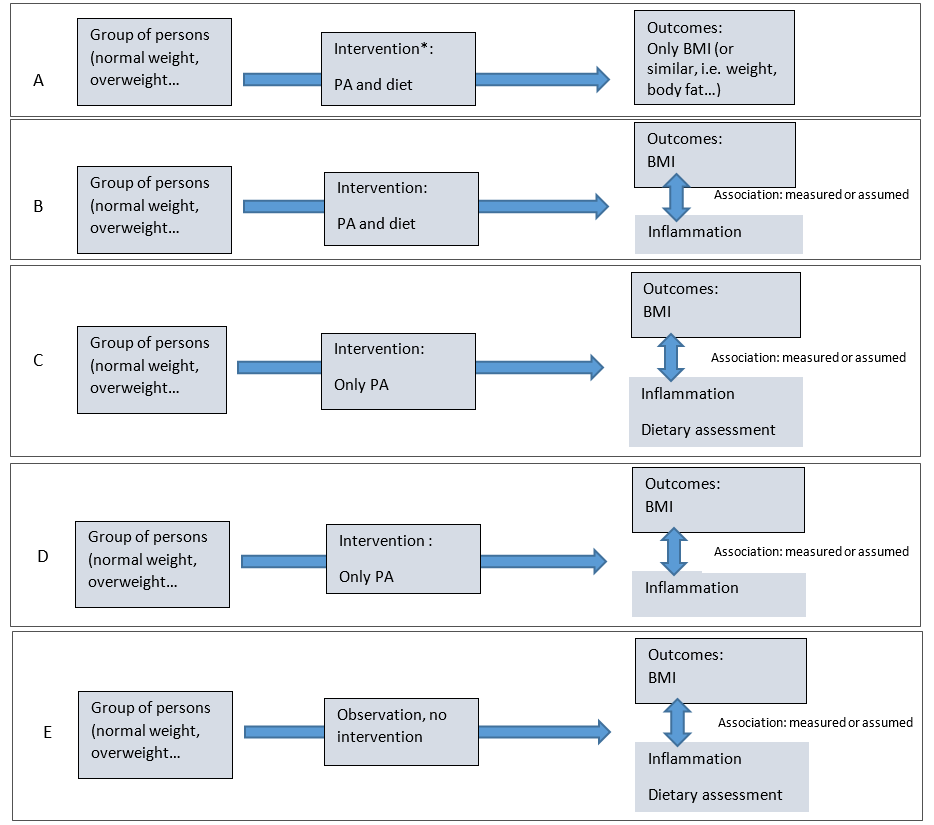
**

**Supplementary Figure 1.** Examples of cases that were considered to be multimodal.

*if listed in the aspects that we were going to investigate, i.e., diet, PA (physical activity), lifestyle factors, etc.


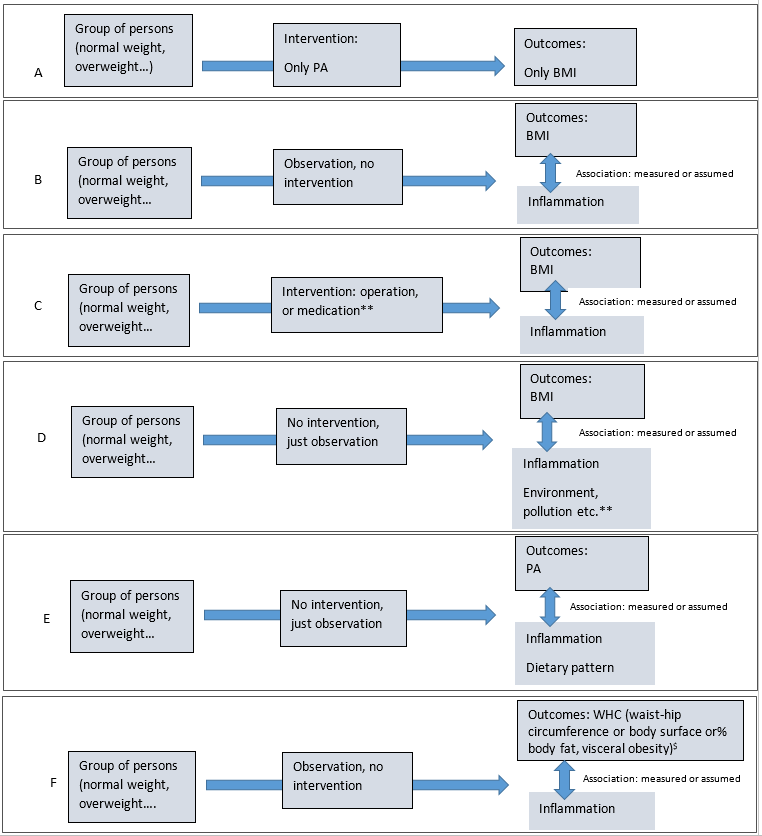


**Supplementary Figure 2**. Examples of cases that were not considered to be multicomponent.

** is an influencing factor, but not one that we investigate here, i.e., it is outside our remit. Drugs and operations were considered rather as treatment and in the medical field.

^$^ is our criterion for obesity instead of BMI (body mass index), i.e., WHC (waist-to-hip ) ratio of >0.9/0.85 (male/female). The same applies to body surface area (a definition exists that accounts for obesity). For body fat, a percentage of>25%/30% (male/female) was considered obesity. For visceral obesity, an area greater than 130 cm² was considered obese. Note: This does not contribute to muscle mass, which is an independent component.

**Supplementary Figure 3.** Temporal distribution of included studies.

| **Supplementary Table 1**. Tier classification approach for articles based on data type relating to the primary outcome and eligible components. | | | | |
| --- | --- | --- | --- | --- |
| **Tier 1** | **Tier 2** | **Tier 3** | **Tier 4** | **Tier 5** |
| Quantitative data relating to the main outcome weight change risk with at least two eligible components (PA, diet, see list…), such as:  - correlations  - regression analyses/associations,  - changes  Could also include meta-analyses/meta-regression | Quantitative data were presented on the main outcome, weight change, and two eligible components (PA, diet…), but no statistical analyses to relate these (correlations, regression analyses), or unsure,  or if weight is related/associated quantitatively with only 1 component  Could include systematic reviews | No quantitative data on either the weight change outcome or any of the at least two components (PA…) are required, such as qualitative descriptions, review articles that merely discuss trends (without presenting data), or scoping reviews without quantitative data. | Article is more hypothetical, such as a new intervention protocol or study protocol. | Does not fit at all (not Tier 1-4), perhaps wrongly selected |
| **Examples***  i. PA intervention, obesity associated with inflammation and gut microbiota,  ii. Observational study, PA (physical activity) patterns related to weight status and gut microbiota,  iii. PA and diet and their combined effect on weight outcome (associations).  * See other examples in the “review protocol and define multicomponent biomarkers” section, **Table 1,** and **Supplementary Figures 1 and 2.** | | | | |

| **Supplementary Table 3.** Overview of the articles in group 4 – diet, omics, and any other outcome | | | | | | | | | |  |
| --- | --- | --- | --- | --- | --- | --- | --- | --- | --- | --- |
| Sub-group | Significant findings in primary outcomes | Total n of articles | Populations | Type of subjects included | Intervention /Exposure description | Main exposure measured | Main outcome | Main outcome measured | References | |
| 1 | 19 | 24 | - adults  - children  - infants  - elderly  - adults & elderly  - children and adults | - healthy  - obese, overweight  - MetS  - CVDs  - increased WC  - elevated serum LDL-C | Interventions  - 3 weeks – 1 year  - 20 – 726 participants  - dietary intervention, supplement intake, probiotics intake  Observation  - 182 – 720 participants  - supplement intake | - diet records  - gut microbiota profiling by sequencing  - fecal SCFA | - BMI  - body fat mass  - WC  - body composition  - waist-hip ratio | - anthropometrics  - DEXA  - BIA | ([216-239](#_ENREF_216)) | |
| 2 | 3 | 3 | - adults  - adults & elderly | - healthy  - obese, overweight | Interventions  - one menstrual cycle – 26 weeks  - 24 - 62 participants  - dietary intervention | - P/B ratio  - AMY1 copy number  - food diaries  - biomarkers of breast cancer  - FFQ  - AHEI, MDS, DASH DASH | - BMI  - WC  - body composition  - body fat mass | - anthropometrics  - DEXA  - BIA | ([244-246](#_ENREF_244)) | |
| 3 | 13 | 16 | - adults  - children  - adults & infants  - adults & elderly | - obese or overweight  - healthy overweight  - obese with prediabetes  - MetS  - healthy | Interventions  - 3 days – 50 weeks  - 23 – 249 participants  - dietary intervention, supplement intake, probiotics intake | - gut microbiota analysis  - diet records | - BMI  - WC  - body fat mass  - body composition  - WHR | - anthropometrics  - DEXA  - BIA  - MRI | ([152](#_ENREF_152), [247-261](#_ENREF_247)) | |
| AHEI: alternative healthy eating index, BIA: bioelectrical impedance analysis, DASH: dietary approaches to stop hypertension, DEXA: dual x-ray absorptiometry, FFQ: food frequency questionnaire, MRI: magnetic resonance imaging, WC: waist circumference, P/B: Proteobacteria (P) and Bacteroidetes (B). | | | | | | | | | | |

| **Supplementary Table 4.** Summary statistics of sample sizes across included studies (n = 296). | | |
| --- | --- | --- |
| N= 554901 | Valid | 292 |
|  | Missing | 0 |
| Mean | | 1900.35 |
| Median | | 93.50 |
| Mode | | 50 |
| Std. Deviation | | 22243.289 |
| Range | | 378867 |
| Minimum | | 10 |
| Maximum | | 378877 |
| Sum | | 554901 |
| Percentiles | 25 | 47.00 |
|  | 50 | 93.50 |
|  | 75 | 286.75 |
